# Role of frailty in predicting outcomes after stroke: a systematic review and meta-analysis

**DOI:** 10.3389/fpsyt.2024.1347476

**Published:** 2024-07-05

**Authors:** Jing Li, Jinping Wan, Hua Wang

**Affiliations:** ^1^ Department of Neurology, People’s Hospital of Anji, Anji County, Huzhou, Zhejiang, China; ^2^ Department of Neurology, Guigang City People’s Hospital, Guigang, Guangxi, China

**Keywords:** frailty, stroke, mortality, poor functional outcome, meta-analysis

## Abstract

**Background:**

Stroke is considered the second most common cause of death and the third leading cause of disability worldwide. Frailty, characterized by increased vulnerability to stressors, is emerging as a key factor affecting outcomes in older adults and stroke patients. This study aimed to estimate the prevalence of frailty in acute stroke patients and assess its association with mortality and poor functional outcome.

**Methods:**

Medline, Google Scholar, and Science Direct databases were systematically searched for English-language studies that included adult stroke patients (>16 years), have defined frailty, and reported mortality and functional outcomes. Meta-analysis was done using STATA 14.2, and the results were expressed as pooled odds ratios (OR) with 95% confidence intervals (CI). Heterogeneity was assessed using the I2 statistic and the Chi-square test. Study quality was evaluated using the Newcastle Ottawa Scale (NOS).

**Results:**

Twenty-five studies were included in the analysis. Frailty prevalence in stroke patients was 23% (95% CI 22% - 23%). Unadjusted analysis showed an OR of 2.66 (95% CI: 1.93 - 3.67) for mortality and 2.04 (95% CI: 1.49 - 2.80) for poor functional outcome. Adjusted estimates indicated an OR of 1.22 (95% CI: 1.1 - 1.35) for mortality and 1.21 (95% CI: 1.04 - 1.41) for poor functional outcome, with substantial heterogeneity for both adjusted and unadjusted analyses. No publication bias was detected for the prevalence of frailty. However, there was a publication bias for the association between frailty and mortality.

**Conclusions:**

Frailty was significantly associated with increased mortality and poorer functional outcomes in stroke patients. Our study highlights the need to focus on frailty in stroke patients to improve outcomes and quality of life. Further research should aim to standardize assessment of frailty and reduce heterogeneity in study outcomes.

**Systematic review registration:**

https://www.crd.york.ac.uk/prospero/#searchadvanced, CRD42023470325.

## Introduction

Stroke is a leading cause of mortality and disability worldwide, presenting a significant public health challenge (1). It is third most common cause of disability (5.7 percent of total disability-adjusted disability years [DALYs]) and the second most common cause of death worldwide (11.6% of total deaths) (2). Stroke often leads to long-term physical, cognitive, and emotional consequences (3) which are associated with a considerable economic burden due to medical costs, lost productivity, and the need for the caregiver support. Predicting the outcomes of stroke is inherently complex due to the heterogeneity of the disease: stroke patients may experience a wide range of clinical symptoms and functional impairments that affect recovery (4). With the continuous aging of the general population, frailty is emerging as a potential key factor in the context of stroke outcomes. Frailty is characterized by multi-systemic decline that impacts the ability of cellular repair mechanisms to maintain system homeostasis, and is linked to increased mortality and higher rates of hospital admissions (5). Several studies have shown that acute stroke patients frequently present with frailty, which is linked to unfavorable outcomes (6, 7).

Many global healthcare systems are currently viewing frailty as an integral component of their acute care pathways (8). While stroke is considered one of the more common acute presentations in older patient, frailty assessment is not yet routinely included in stroke care, and stroke is rarely mentioned in best practice guidelines on frailty (9). Studies, evaluating the impact of frailty on the outcomes in stroke patients are still scarce. Previous existing meta-analyses by Bao et al. (2023), and Burton et al. (2022) included only eight and 14 studies, respectively, with an literature search up to 2020 (10, 11). While a recent study by Huang et al. updated the existing body of evidence, it focused exclusively on the burden of frailty in stroke patients (12). There is no existing research that has determined the association between frailty and mortality in this vulnerable group. This study aims to summarize the existing data, update a baseline estimation on the prevalence of frailty in stroke patients, and to assess the link between frailty and stroke outcomes.

## Materials and methods

### Research questions

To estimate the prevalence of frailty in patients with acute stroke and to assess the association between frailty and clinical outcomes (mortality and poor functional outcome) in this population.

### Methods

Literature search was done in Medline, Google Scholar and Science Direct databases from inception till October 2023 for articles published in English language. Data screening was performed independently by the two reviewers, and all disagreements were resolved by the principal investigator (PI). The review was reported in accordance with the latest “Preferred reporting items for systematic reviews and meta-analyses (PRISMA)” framework (13). The data extraction template was prepared by the PI, who also double checked the data entry for correctness. Ethical approval is not applicable since we extracted data from freely available sources.

### Inclusion and exclusion criteria

#### Population

Adult patients (>16 years of age) who presented with any type of stroke (except for transient ischemic attacks) were included. Only patients who presented with acute stroke were included, while patients who were undergoing rehabilitative therapy were excluded.

#### Exposure

The exposure of interest was frailty. We accepted any recommended definition of frailty used by individual studies if that have quantified or categorized frailty levels and have confirmed its onset before the stroke event. In addition, we included studies that reported prevalence of frailty among patients with acute stroke.

#### Outcome

Our primary outcome of interest was mortality (reported at various time points – in hospital to 1 year follow up as reported by the studies), functional outcome and length of hospital stay.

#### Study design

We included all analytical studies (prospective, retrospective, and cross-sectional studies).

### Search strategy

We utilized Medical subject heading (MeSH) terms such as: “Stroke” OR “Cerebrovascular accident” AND “Fragility” OR “Elderly” AND “Mortality” OR “Death” OR “Survival” AND “functional outcome” AND “Observational studies” OR “Cohort studies” OR “Prospective studies”. The references of included studies for potentially eligible reports. The detailed search strategy is explained in **Supplementary File 1**.

### Data extraction and management

The first and the second authors independently extracted data such as author’s details, study design, sample size, geographical location, inclusion criteria, definition of frailty and classification tools used to assess it.

### Statistical analysis

STATA 14.2. was used for analysis. Inverse variance method was used for binary outcomes to combine the effects across various studies. The outcome was then expressed as pooled odds ratios (OR) with 95% confidence intervals (CIs). For each study reporting prevalence of frailty among patients with diagnosed stroke, standard error was computed by using provided prevalence and sample size. To perform the prevalence meta-analysis, we employed the “metaprop” function (14). To account for the potential influence of both large and small studies on the pooled estimates, we applied the Freeman-Tukey double arcsine transformation. The final pooled prevalence was reported, along with its corresponding 95% CI. In cases of missing data, attempts were made to contact the respective authors for the necessary information. The results were presented as pooled effect sizes and visually depicted through forest plots. Funnel plots and Egger’s test (15) were used to assess publication bias. P<0.05 indicated statistical significance.

Variability between studies was assessed by I2 statistic and Chi-square heterogeneity test. We categorized heterogeneity into three levels: mild (I2 < 25%), moderate (I2 between 25% and 75%), and considerable (I2 > 75%).

### Quality assessment of included studies

Quality of the studies included in our analysis was evaluated by the Newcastle Ottawa Scale (NOS) (16). This scale assesses study quality based on three criteria: ascertainment of outcomes, selection of study groups, and comparability. In the Selection and Outcome categories, a study can receive a maximum of one star for each numbered item. For comparability, a maximum of two stars can be assigned. Therefore, the NOS allows for a maximum score of nine for each study.

## Results

### Study selection

**Figure 1:**
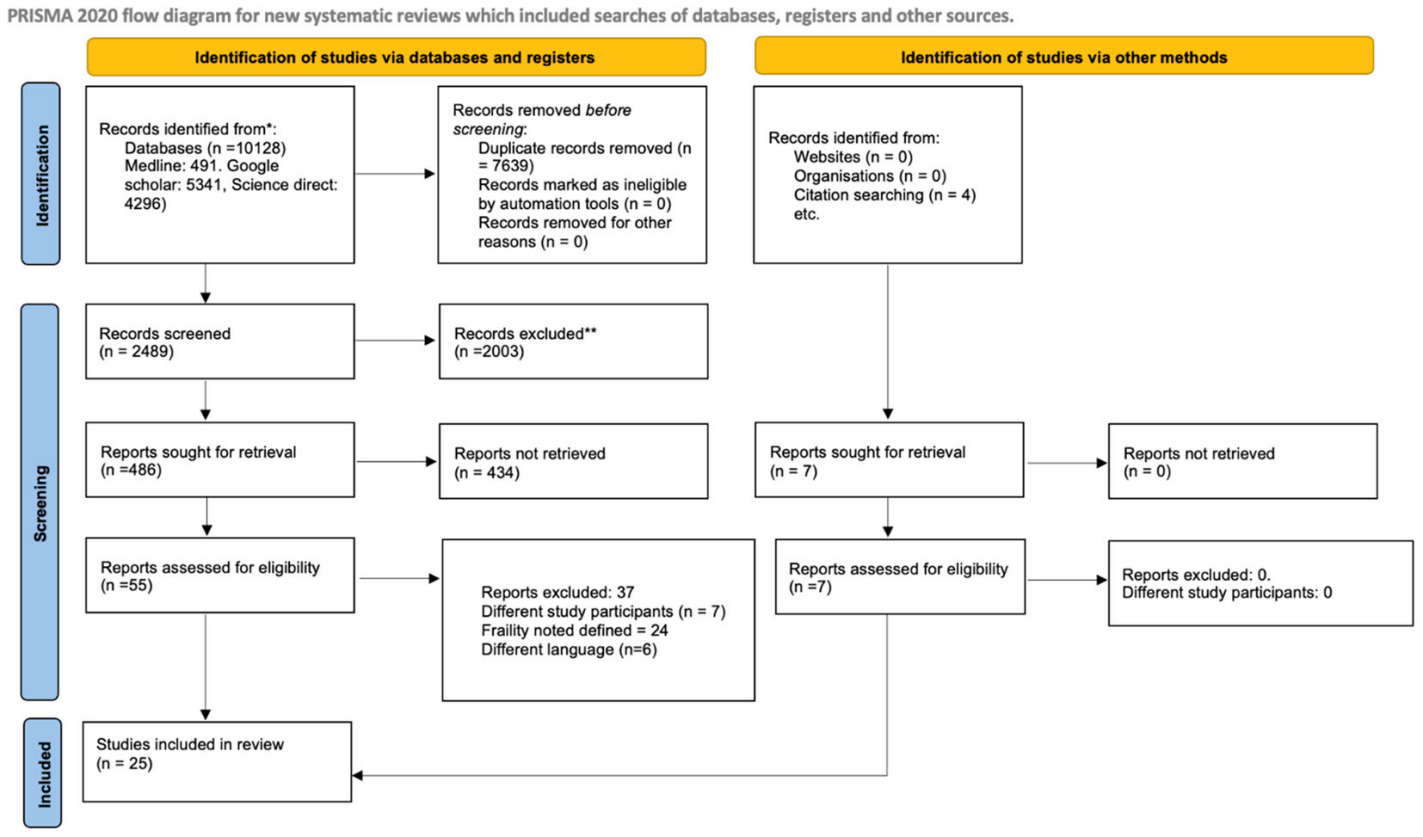
PRISMA 2020 flow diagram explaining the Search flow.

A total of 10128 articles were identified by the literature search. After primary screening, 7639 papers were removed as duplicates. Additional 2003 studies were eliminated at the stage of title and abstract screening. From the remaining 486 studies, 55 free full text articles were retrieved. Finally, 25 articles that met eligibility criteria were included in our systematic review and meta-analysis (17–41). Twenty articles reported on the prevalence of frailty among stoke patients, 17 reported on the association between mortality and frailty, and eight studies reported the association between poor functional outcome and frailty. The PRISMA 2020 flow diagram of the study is explained in **Figure 1**.

### Characteristics of the included studies

General characteristics of the included studies are detailed in **Table 1**. Of 25 included studies, 12 were from Europe, eight were from Asia, three from America and two from Australia. Included studies had sample sizes ranging from 102 to 48980. All studies reported results in English language. Studies defined frailty based on different assessment tools, with the most commonly used tool being Clinical Frailty Scale (CFS).

**Table 1:** Characteristics of included studies, n=25.

Study	Continent	Sample size	Study type	Definition of Frailty	Tool used	Type of stroke	Severity – Scale (Median or %)	Age (median and range/Mean (SD))	Inclusion and Exclusion criteria	Quality of study (NOS)
Rowan 2023 (17)	Europe	332	Prospective longitudinal cohort study	Frailty defined by FI score of ≥0.24	33-item Frailty index (FI)	Ischaemic Stroke	NIHSS 2 (1–4)	71 (60–80)	Stroke and transient ischaemic attack (TIA) survivors admitted to participating acute stroke centres (2016 to 2019) with no exclusions	8
Zhang 2022 (18)	Asia	530	Prospective longitudinal cohort study	Frailty scores (3–5)	FRAIL scale	Ischaemic Stroke	Not provided	72.94 ± 5.79	Patients with abrupt onset of focal neurologic deficits with clinical symptoms with head CT scans showing haemorrhage or early signs of infarction.	9
Ng 2023 (19)	Asia	384	Retrospective observational cohort study	Frailty (CFS = 5–8)	CFS score	Not specified	Not provided	81.7 ± 6.5	All older adults aged ≥65 years admitted to the centre with acute stroke between 2016 2020	8
Varquez 2023 (20)	America	48980	Cross sectional descriptive analysis	3 or above from the mF-11	mF-11	Lacunar stroke	NIHSS score >20 (0.4%)	Not provided	Not provided	7
Hanlon 2023 (21)	Europe	9324	Cross sectional analysis	Frailty (CFS = 5–8)	Clinical Frailty Scale (CFS)	Not specified	Not provided	Not provided	Adult patients with acute stroke	8
Yang 2022 (22)	Asia	205	Prospective cohort study	Frailty scores (3–5)	FRAIL scale	Not specified	NIHSS 3 (1–7)	74(70–80)	Patients between 65 - 99 years with acute cerebral infarction attending the centre from 2019 to 2020.	6
Nozoe 2022 (23)	Asia	317	Prospective cohort study	Frail >0.24	33-item Frailty Index	Stroke and intracerebral haemorrhage	NIHSS 2 (3)	76 (12)	Patients aged ≥65 years who were diagnosed with cerebral infarction or intracerebral haemorrhage were included	8
Miranda 2022 (24)	America	174	Prospective cohort study	PRISMA-7 (cutoff > =3)	PRISMA 7	Ischaemic Stroke	NIHSS 5.0 (2.0 to 9.0)	69	Participants more than 40 years who suffered an acute ischemic stroke that was confirmed by neuroimaging and a stroke neurologist.	7
Noguchi 2021 (25)	Asia	232	Prospective longitudinal study	Not defined	NIHSS	Ischaemic Stroke	NIHSS 2 (3)	76 (11)	Older stroke patients aged 65 years or older who were admitted to the centre between 2017 and 2019	7
Kilkenny 2021 (26)	Australia	15468	Observational study	High risk >15	Hospital Frailty Risk Score	Ischaemic Stroke	Not provided	67.0 (57.4–75.6)	All patients with stroke (ischemic, intracerebral haemorrhage) or TIA, aged ≥18 years	9
Joyce 2022 (27)	Europe	175	Cross sectional descriptive analysis	Frailty defined by FI score of ≥0.24	33-item Frailty index (FI)	Ischaemic Stroke	NIHSS 19 (15–24)	80 (7.7)	Adult patients with acute stroke	8
Pinho 2021 (28)	Europe	489	Retrospective study	High risk >15	Hospital Frailty Risk Score	Ischaemic Stroke	NIHSS 16 (12–20)	78 (70–84)	Adult patients with acute stroke	7
Schnieder 2021 (29)	Europe	318	Retrospective observational cohort study	High risk >15	Hospital Frailty Risk Score	Ischaemic Stroke	NIHSS 15 (10)	80.1 (9.58)	Elderly patients ≥ 65 years being admitted with large vessel occlusion stroke (LVOS) and endovascular treatment.	6
Waehler 2021 (30)	Europe	625	Prospective cohort study	Frail 3 or more Fried criteria	Fried Frailty Phenotype	Ischaemic Stroke	NIHSS 2.8 (4)	71.7 (11.6)	Adult patients with acute stroke	9
Zhang 2020 (31)	Australia	2098	Observational, cohort study	High risk >15	Hospital Frailty Risk Score	Haemorrhagic stroke	Not provided	76 (65, 84)	Adult patients with acute stroke	8
Kanai 2020 (32)	Asia	234	Cross-sectional study	Frailty > = 3	5-item Simplified frailty index	Ischaemic Stroke	NIHSS 1 (0–3)	73.0 (68.8–78.0)	Elderly patients with stroke within 48 hours of stroke symptom onset	7
Evans 2020 (33)	Europe	433	Cohort study	Frailty (CFS = 5–8)	Clinical Frailty Scale (CFS)	Ischaemic Stroke	NIHSS	83 (77–86)	Individuals aged >75 years with ischaemic stroke at the centre between 2013 and 2016	8
Myint 2017 (34)	Europe	2388	Cohort study	Not defined	Pre-stroke modified Rankin scale	Ischaemic Stroke	Not provided	76.9 (12.7)	Adult patients with acute stroke	9
Seamon 2019 (35)	America	7258	Retrospective cohort	Frail >5.0	Faurot Frailty Index	Ischaemic Stroke	Stroke administrative severity index	79.4 (8.4)	Adult patients with acute stroke	8
Rowan 2019 (36)	Europe	154	Cohort study	Frail >0.24	33-item Frailty Index	Ischaemic Stroke	Not provided	69.3 (7.2)	Adult patients with acute stroke	8
Tiainen 2022 (37)	Europe	159	Cross sectional analysis	Frailty (CFS = 5–8)	Clinical Frailty Scale (CFS)	Ischaemic Stroke	NIHSS 12 (6–19)	83 (81–86)	Patients aged >80 years with acute stroke	7
Tan 2022 (38)	Asia	198	Retrospective cohort study,	Frailty (CFS = 5–8)	Clinical Frailty Scale (CFS)	Ischaemic Stroke	NIHSS 14 (10–20)	75.7 (4.3)	Acute Ischemic Stroke patients age≥70 years reported to the centre from 2017 to 2020.	8
Patel 2022 (39)	Europe	472	Cohort study	Frailty (CFS = 5–8)	Clinical Frailty Scale (CFS)	Ischaemic Stroke	NIHSS Not provided	79 (69–86)	Patient diagnosed with a stroke by a consultant stroke physician clinically, with imaging (CT)	7
Pilotto 2022 (41)	Europe	102	Cohort study	3 or above from the mF-11	mF-11	Not specified	Not provided	77.5 (65–94)	Consecutive patients older than 65 years who underwent reperfusion treatment in a single stroke unit from 2015 to 2016	6
Imaoka 2018 (40)	Asia	156	Retrospective analysis	3 or above from the mF-11	mF-11	Haemorrhagic stroke	Not provided	66.0 ± 13.4	Adult patients with acute stroke	6

### Excluded studies

Of 55 full-text articles that were retrieved, 37 were excluded during the secondary screening. Of them, 24 studies did not define frailty, six were reported in languages other than English, and seven had mixed study population.

### Burden of frailty among stroke patients

**Figure 2:**
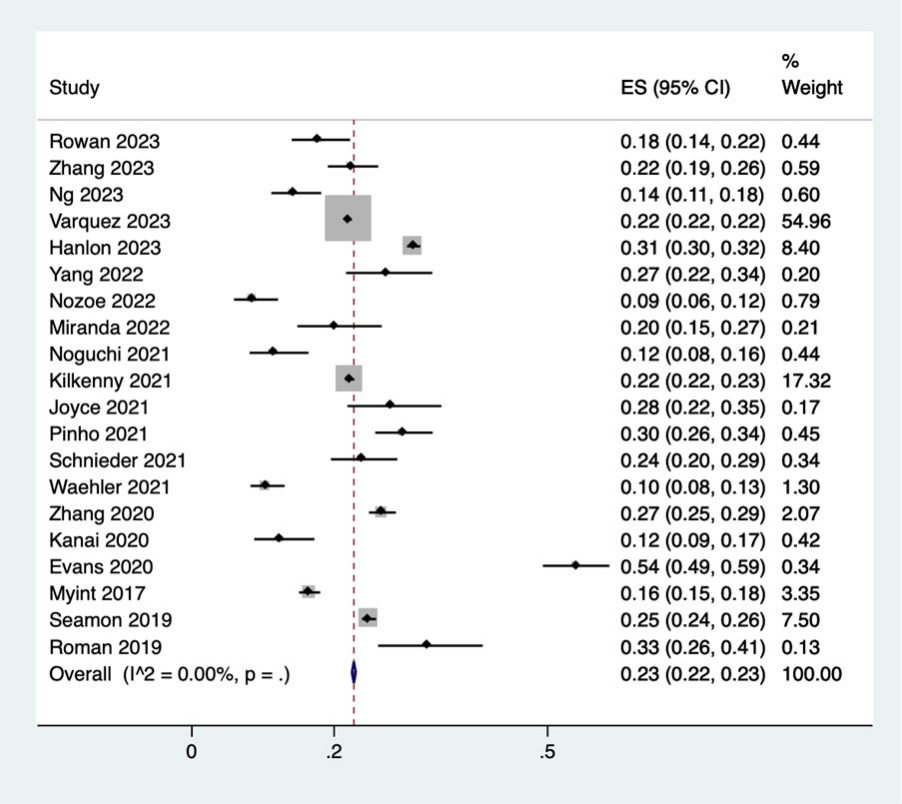
Forest plot showing the prevalence of frailty among stroke patients.

Prevalence of frailty in stroke patients was reported in 20 studies (n=90118). After adjusting for population weights, the overall pooled prevalence of frailty in stroke patients was 23% (95%CI 22% - 23%) with no heterogeneity (I2 = 0.0). (**Figure 2**) A subgroup analysis analyzing the difference in prevalence estimates with respect to region of the included studies showed that the prevalence was lower in studies from the Asian continent (15%, 95%CI 13% - 16%), (**Supplementary File 2**).

### Association between frailty and mortality among stroke patients: (unadjusted estimates)

**Figure 3:**
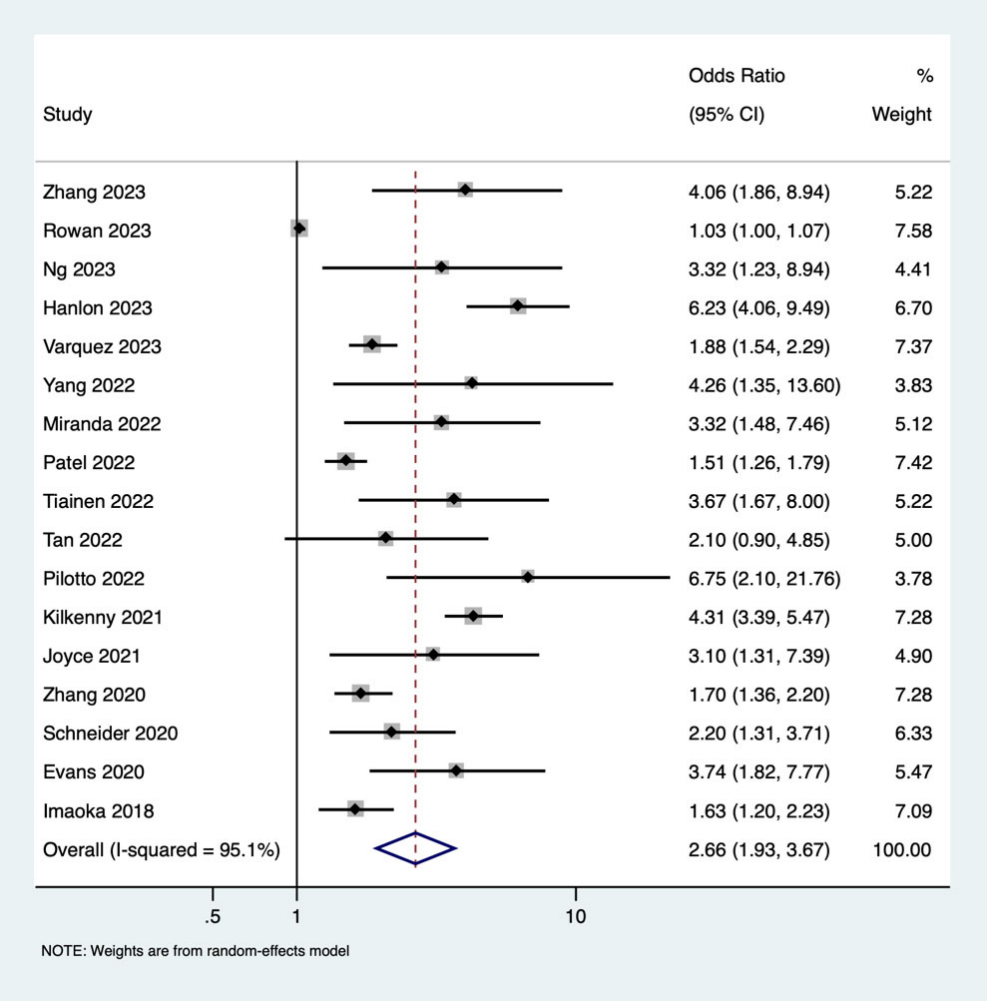
Forest plot showing the association between frailty and mortality (unadjusted).

**Figure 4:**
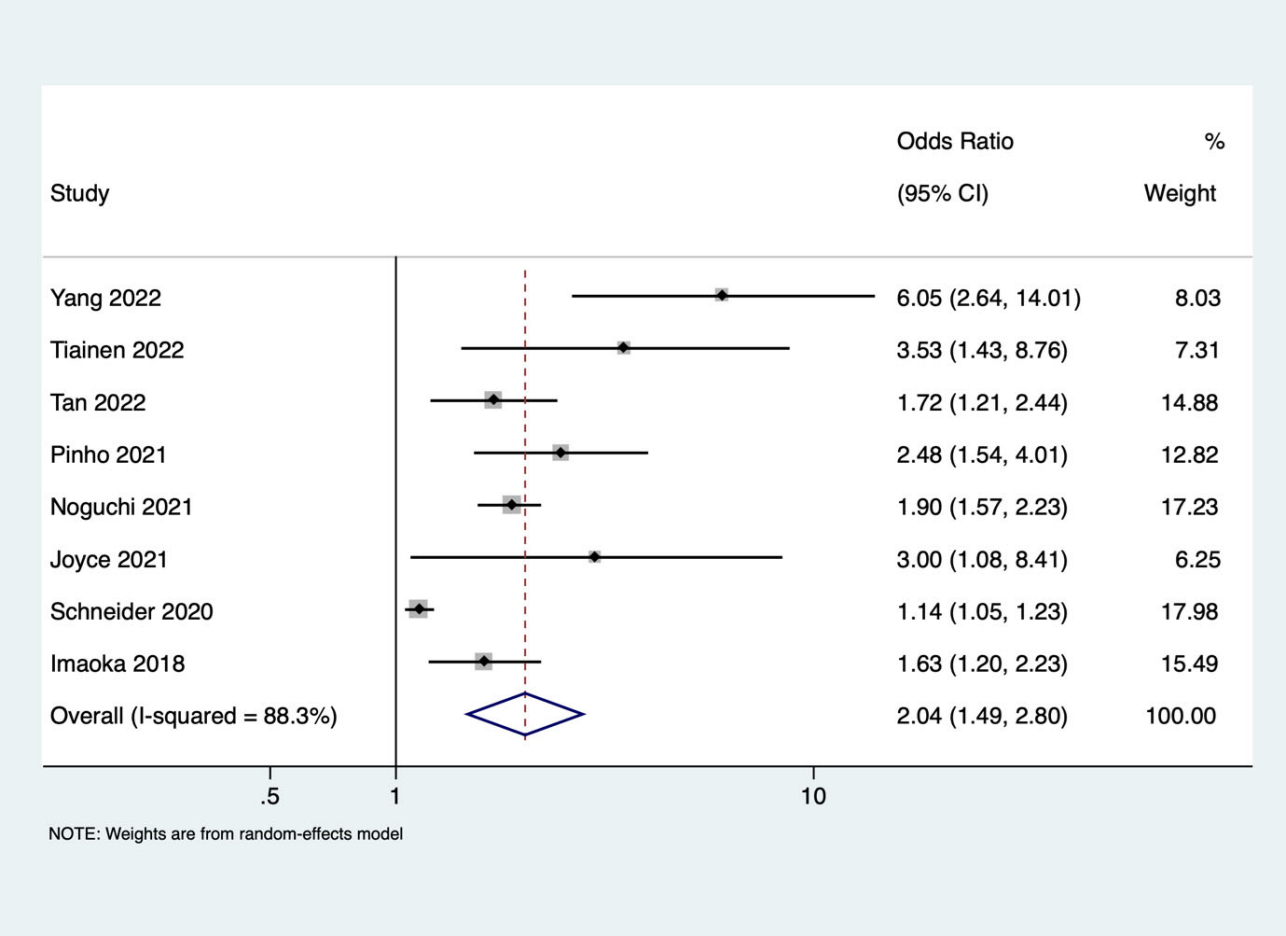
Forest plot showing the association between frailty and poor functional outcome (unadjusted).

Seventeen studies reported the unadjusted association between frailty and mortality. We saw that patients with frailty and stroke had 2.66 higher odds of mortality when compared to stroke patients without frailty (pooled OR of 2.66, 95% CI: 1.93 - 3.67), with very high heterogeneity (I^2^ = 95.1, p-value <0.001). (**Figure 3**) Eight studies reported on the unadjusted association between frailty and poor functional outcome. Frail patients had 2.04-higher odds of having poor functional outcome when compared to patients without frailty (pooled OR of 2.04, 95% CI: 1.49 - 2.80), with high heterogeneity (I^2^ = 88.3, p value <0.001). (**Figure 4**) Subgroup analysis based on the region of included studies for mortality and poor functional outcome is shown in **Supplementary File 3** and **Supplementary File 4** respectively.

### Association between frailty and mortality among stroke patients: (adjusted estimates)

Eleven out of seventeen studies that reported on association between frailty and mortality reported adjusted estimates. After adjusting for potential confounders, our analysis demonstrated that frailty in stroke patients was associated with 1.22-higher odds of mortality when compared to stroke patients without frailty (pooled OR of 1.22, 95% CI: 1.1 - 1.35), with high heterogeneity (I^2^ = 83.3, p-value <0.001). (**Supplementary File 5**) Six out of eight studies reported on the adjusted association between frailty and poor functional outcome. The pooled evidence showed that after adjusting for potential confounders, frailty was associated with 1.21-higher odds of ending up with poor functional outcomes (pooled OR of 1.21, 95% CI: 1.04 - 1.41, with high heterogeneity I^2^ = 80.8, p value <0.001). (**Supplementary File 6**). **Supplementary Files 7** and **8** describes the subgroup analysis based on the region of included studies for mortality and poor functional outcome.

### Publication bias

**Figure 5:**
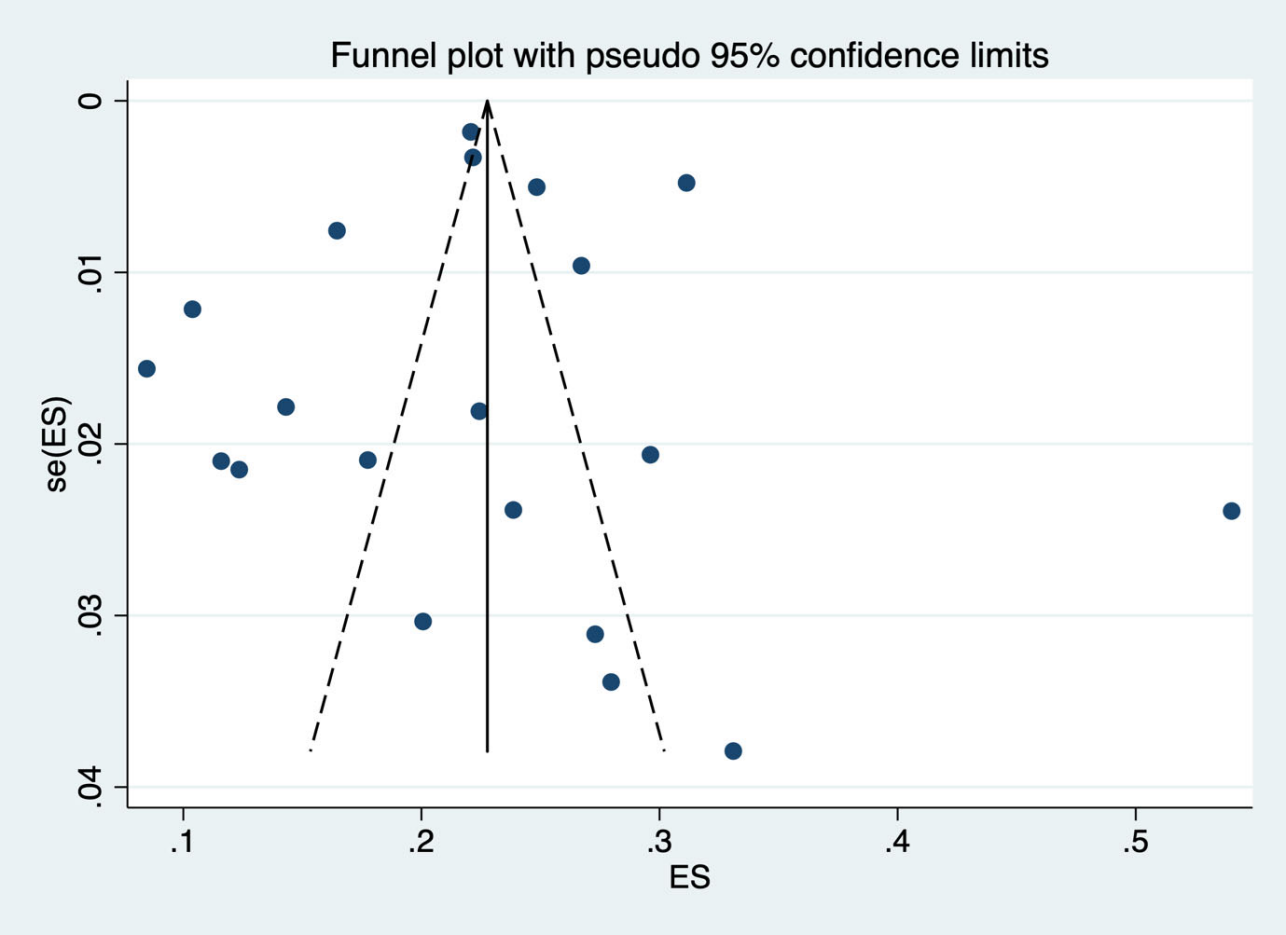
Funnel plot showing publication bias for studies reporting prevalence.

We evaluated the presence of publication bias for prevalence and the association between frailty and mortality (both adjusted and unadjusted) as they had more than 10 studies. We noted that the funnel plots were symmetrical for prevalence estimates, confirming the absence of publication bias (Egger coefficient -0.23, p value 0.91). (**Figure 5**) However, we observed clear asymmetry in funnel plot for both unadjusted (**Supplementary File 9**), and adjusted estimates (**Supplementary File 10**) for association between frailty and mortality.

### Risk of bias in included studies

As shown in **Table 1**, most included studies had low risk of bias, with the NOS scores of 6 to 8. The difference in effect estimates for various outcomes with respect to risk of bias scores is expressed in **Supplementary Files 11**-**15**. Subgroup analysis based on the tool used is described in **Supplementary File 16**. We did not detect any difference in the mortality prevalence, except for three studies that had used Faurot frailty index, pre stroke modified Rankin scale, 5 item simplified frailty scale, and frailty phenotype – all of which showed mortality less than 15%. Comparison of the length of hospital stay in patients with and without frailty is described in **Supplementary File 17**. Subgroup analyses based on the study design of the included studies for unadjusted mortality estimates are included in **Supplementary File 18**.

## Discussion

Our meta-analysis that included 25 studies showed that the prevalence of frailty among patients with stroke was 23% (95%CI 22% - 23%). Our results detected a significant association between frailty and mortality (adjusted OR 1.22, 95% CI: 1.1 - 1.35) and poor functional outcome (adjusted OR 1.21, 95% CI: 1.04 - 1.41) even after adjusting for potential confounders. The findings of our review further emphasize the importance of frailty assessment in predicting outcomes after stroke.

Our results showed that the pooled prevalence of frailty in stroke patients was 23 percent, as reported by 20 studies from various study settings. This is consistent with the growing understanding that frailty is a common problem among elderly stroke patients (11, 12). Previous studies have shown that factors such as type of assessment method and geographic location can affect the prevalence of frailty (42). This is supported by our subgroup analysis, which reveals that the prevalence of frailty in Asian studies was 15% lower than that in non-Asian populations.

Our results demonstrated that stroke patients with frailty are at significantly higher risk of mortality compared to patients without frailty (OR of 2.66). Moreover, after accounting for potential confounders, frailty remained independently associated with increased mortality in stroke patients (OR of 1.22). These findings are consistent with the previous reports (11, 37) which further strengthen the prognostic significance of frailty in stroke patients. The observed high heterogeneity in both unadjusted and adjusted estimates underscores the need for further investigation into factors contributing to this variability (43).

Both unadjusted and adjusted estimates in our study showed that frailty was significantly associated with poor functional outcomes post-stroke. These results have crucial implications for rehabilitation and allocation of resources to support functional recovery of stroke survivors.

In Europe, the prevalence of frailty in persons 65 years of age or older is currently estimated to be over 15% (42), rising to over 25% (44) in adults 85 years of age or older. These estimates are in line with our subgroup analysis findings. Studies showed that clinical frailty positively correlated with stroke severity in older patients and with 28-day mortality following ischemic stroke (45). We may speculate that these findings can be partially explained by the multifaceted nature of frailty. The term “frailty” refers to a range of psychological, social, and physiological characteristics that, together with stroke-related factors, may affect outcomes of stroke patients. Frailty is defined physiologically as a reduced reserve and increased susceptibility to stressors. In stroke patients, these stressors include the cerebrovascular event itself and subsequent complications. Reduced physiological resilience of frail patients may, therefore, translate into poorer outcomes. Frailty is often accompanied by psychological, cognitive and emotional challenges that can worsen after the stroke, impairing self-care and rehabilitation compliance. Additionally, while social networks are essential for stroke rehabilitation, their availability may be restricted by frailty. Stroke patients with frailty may, therefore, face challenges in accessing caregiving resources and rehabilitation services, potentially affecting the effectiveness of their recovery.

### Clinical implications

Our results have substantial clinical implications. By recognizing frailty as a key predictor of stroke outcomes, clinicians can refine risk stratification and individualize care plans. Early interventions targeting frailty, such as physical therapy, nutrition support, and psychosocial interventions, can be integrated into stroke management protocols. These measures may improve post-stroke recovery and reduce the burden of frailty-related complications. Moreover, policymakers and healthcare systems should consider the inclusion of frailty assessments in stroke care guidelines. This could lead to improved resource allocation, including the provision of rehabilitation services and social support tailored to the needs of frail stroke survivors.

### Strengths and limitations

Our review is one of the few studies that attempted to find the link between frailty and stroke outcomes. The increased power of our review due to the large sample size is another major strength. We have also assessed the risk of publication bias and showed that our summary estimates were robust across various subgroup and sensitivity analyses. However, our study has several limitations. First, most included studies were observational with a retrospective design, which might have biased our results. Second, we were unable to adjust for all confounding variables. Finally, not all studies used the same frailty assessment tools, resulting in differences in estimates and high clinical heterogeneity. Additionally, we could not completely exclude the possibility of language and publication bias.

### Conclusions

In conclusion, this systematic review and meta-analysis showed that approximately fifth of older stroke patients are frail, which could potentially influence their clinical outcome. Our study underscores the importance of addressing frailty in the context of stroke. By recognizing and managing frailty, healthcare providers and policymakers have an opportunity to improve outcomes and quality of life of this vulnerable population. Future studies should aim to reduce heterogeneity by standardizing frailty assessment. We recommend incorporation of frailty assessment in elderly stroke management. Clinicians should consider specific rehabilitation interventions targeting nutritional and psychosocial needs, based on the frailty status of stroke survivors.

## Data availability statement

The original contributions presented in the study are included in the article/**Supplementary Materials**. Further inquiries can be directed to the corresponding author.

## Author contributions

JL: Conceptualization, Data curation, Investigation, Supervision, Writing – original draft, Writing – review & editing. JW: Conceptualization, Data curation, Formal analysis, Investigation, Methodology, Validation, Writing – original draft. HW: Conceptualization, Data curation, Formal analysis, Funding acquisition, Investigation, Resources, Supervision, Validation, Visualization, Writing – original draft, Writing – review & editing.
