# Supplementary material for: Role of frailty in predicting outcomes after stroke: a systematic review and meta-analysis
Source: Front Psychiatry. 2024 Jul 5;15:1347476. doi: 10.3389/fpsyt.2024.1347476 (PMC11257970; doi:10.3389/fpsyt.2024.1347476)

Supplementary file 1: Search strategy

**SEARCH STRATEGY: PUBMED**	
S.NO	SEARCH TERMS
#1	Search: **((frailty) AND (stroke)) AND (mortality)**<br>("frailty"[MeSH Terms] OR "frailty"[All Fields] OR "frailties"[All Fields]) AND ("stroke"[MeSH Terms] OR "stroke"[All Fields] OR "strokes"[All Fields] OR "stroke s"[All Fields]) AND ("mortality"[MeSH Terms] OR "mortality"[All Fields] OR "mortalities"[All Fields] OR "mortality"[MeSH Subheading])<br>**Translations**<br>**frailty:** "frailty"[MeSH Terms] OR "frailty"[All Fields] OR "frailties"[All Fields]<br>**stroke:** "stroke"[MeSH Terms] OR "stroke"[All Fields] OR "strokes"[All Fields] OR "stroke's"[All Fields]<br>**mortality:** "mortality"[MeSH Terms] OR "mortality"[All Fields] OR "mortalities"[All Fields] OR "mortality"[Subheading]
#2	Search: **((frailty) AND (stroke)) AND (poor functional outcome)**<br>("frailty"[MeSH Terms] OR "frailty"[All Fields] OR "frailties"[All Fields]) AND ("stroke"[MeSH Terms] OR "stroke"[All Fields] OR "strokes"[All Fields] OR "stroke s"[All Fields]) AND (("poverty"[MeSH Terms] OR "poverty"[All Fields] OR "poor"[All Fields]) AND ("functional"[All Fields] OR "functional s"[All Fields] OR "functionalities"[All Fields] OR "functionality"[All Fields] OR "functionalization"[All Fields] OR "functionalizations"[All Fields] OR "functionalize"[All Fields] OR "functionalized"[All Fields] OR "functionalizes"[All Fields] OR "functionalizing"[All Fields] OR "functionally"[All Fields] OR "functionals"[All Fields] OR "functioned"[All Fields] OR "functioning"[All Fields] OR "functionings"[All Fields] OR "functions"[All Fields] OR "physiology"[MeSH Subheading] OR "physiology"[All Fields] OR "function"[All Fields] OR "physiology"[MeSH Terms]) AND ("outcome"[All Fields] OR "outcomes"[All Fields]))<br>**Translations**<br>**frailty:** "frailty"[MeSH Terms] OR "frailty"[All Fields] OR "frailties"[All Fields]<br>**stroke:** "stroke"[MeSH Terms] OR "stroke"[All Fields] OR "strokes"[All Fields] OR "stroke's"[All Fields]<br>**poor:** "poverty"[MeSH Terms] OR "poverty"[All Fields] OR "poor"[All Fields]<br>**functional:** "functional"[All Fields] OR "functional's"[All Fields] OR "functionalities"[All Fields] OR "functionality"[All Fields] OR "functionalization"[All Fields] OR "functionalizations"[All Fields] OR "functionalize"[All Fields] OR "functionalized"[All Fields] OR "functionalizes"[All Fields] OR "functionalizing"[All Fields] OR "functionally"[All Fields] OR "functionals"[All Fields] OR "functioned"[All Fields] OR "functioning"[All Fields] OR "functionings"[All Fields] OR "functions"[All Fields] OR "physiology"[Subheading] OR "physiology"[All Fields] OR "function"[All Fields] OR "physiology"[MeSH Terms]<br>**outcome:** "outcome"[All Fields] OR "outcomes"[All Fields]
#1<br>OR<br>#2	Search: **((frailty) AND (stroke)) AND ((poor functional outcome) OR (Mortality))**<br>("frailty"[MeSH Terms] OR "frailty"[All Fields] OR "frailties"[All Fields]) AND ("stroke"[MeSH Terms] OR "stroke"[All Fields] OR "strokes"[All Fields] OR "stroke s"[All Fields]) AND ((("poverty"[MeSH Terms] OR "poverty"[All Fields] OR "poor"[All Fields]) AND ("functional"[All Fields] OR "functional s"[All Fields] OR "functionalities"[All Fields] OR "functionality"[All Fields] OR "functionalization"[All Fields] OR "functionalizations"[All Fields] OR "functionalize"[All Fields] OR "functionalized"[All Fields] OR "functionalizes"[All Fields] OR "functionalizing"[All Fields] OR "functionally"[All Fields] OR "functionals"[All Fields] OR "functioned"[All Fields] OR "functioning"[All Fields] OR "functionings"[All Fields] OR "functions"[All Fields] OR "physiology"[MeSH Subheading] OR "physiology"[All Fields] OR "function"[All Fields] OR "physiology"[MeSH Terms]) AND ("outcome"[All Fields] OR

	"outcomes"[All Fields])) OR ("mortality"[MeSH Terms] OR "mortality"[All Fields] OR "mortalities"[All Fields] OR "mortality"[MeSH Subheading]))<br>**Translations**<br>**frailty:** "frailty"[MeSH Terms] OR "frailty"[All Fields] OR "frailties"[All Fields]<br>**stroke:** "stroke"[MeSH Terms] OR "stroke"[All Fields] OR "strokes"[All Fields] OR "stroke's"[All Fields]<br>**poor:** "poverty"[MeSH Terms] OR "poverty"[All Fields] OR "poor"[All Fields]<br>**functional:** "functional"[All Fields] OR "functional's"[All Fields] OR "functionalities"[All Fields] OR "functionality"[All Fields] OR "functionalization"[All Fields] OR "functionalizations"[All Fields] OR "functionalize"[All Fields] OR "functionalized"[All Fields] OR "functionalizes"[All Fields] OR "functionalizing"[All Fields] OR "functionally"[All Fields] OR "functionals"[All Fields] OR "functioned"[All Fields] OR "functioning"[All Fields] OR "functionings"[All Fields] OR "functions"[All Fields] OR "physiology"[Subheading] OR "physiology"[All Fields] OR "function"[All Fields] OR "physiology"[MeSH Terms]<br>**outcome:** "outcome"[All Fields] OR "outcomes"[All Fields]<br>**Mortality:** "mortality"[MeSH Terms] OR "mortality"[All Fields] OR "mortalities"[All Fields] OR "mortality"[Subheading]
**SEARCH STRATEGY: SCIENCE DIRECT**	
#1	**((frailty) AND (stroke)) AND ((poor functional outcome) AND (Mortality))**
**SEARCH STRATEGY: GOOGLE SCHOLAR**	
#1	Frailty; Stroke; Transient ischemic attack; Poor functional outcome; Mortality; Cohort studies; Case-control studies; Cross-sectional studies; Observational studies

Supplementary file 2: Forest plot showing the prevalence of frailty among stroke patients grouped by continent

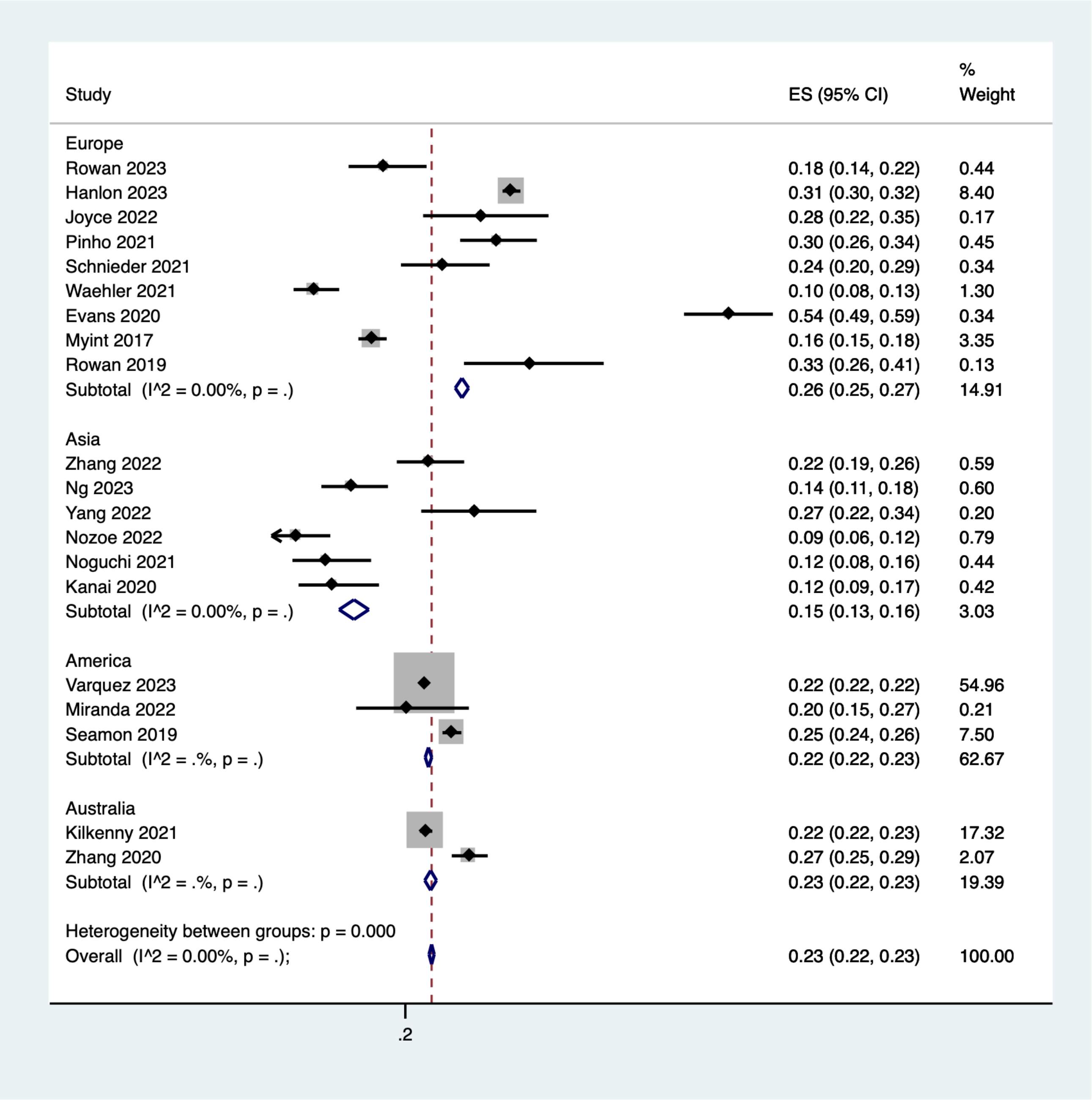

Supplementary file 3: Forest plot showing the association between frailty and mortality among stroke patients grouped by continent (unadjusted)

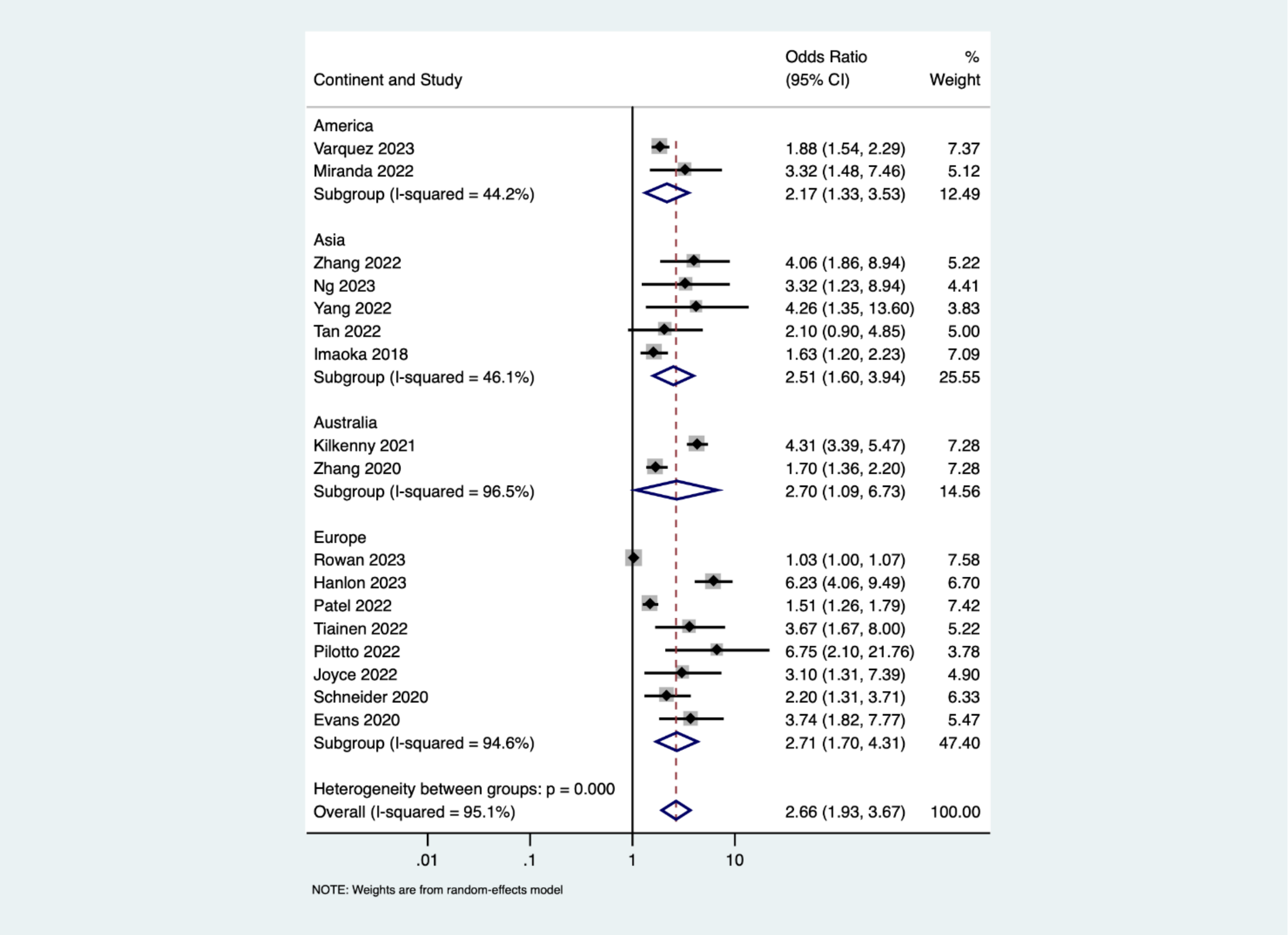

Supplementary file 4: Forest plot showing the association between frailty and poor functional outcome among stroke patients grouped by continent (unadjusted)

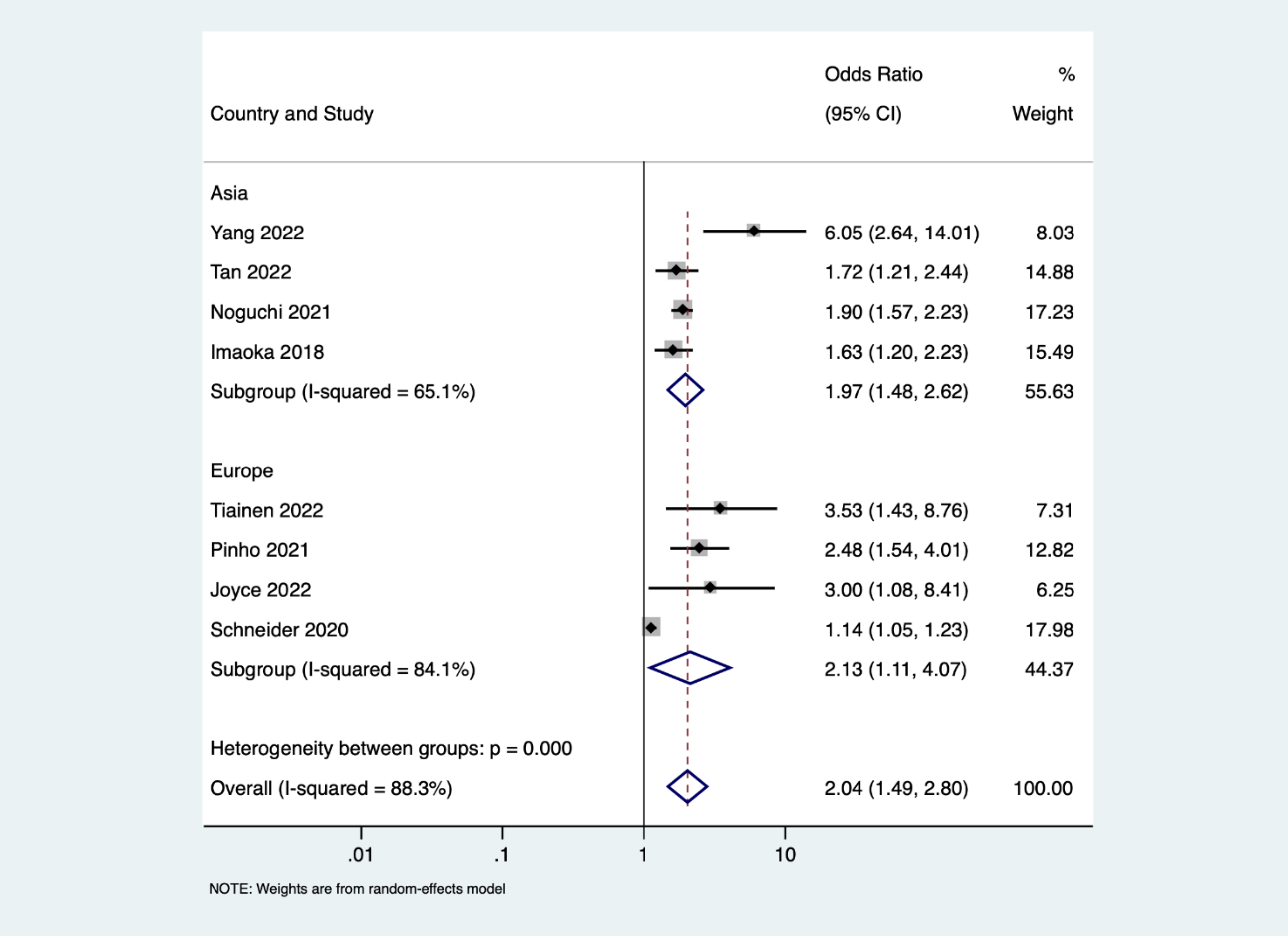

Supplementary file 5: Forest plot showing the association between frailty and mortality among stroke patients grouped by continent (adjusted)

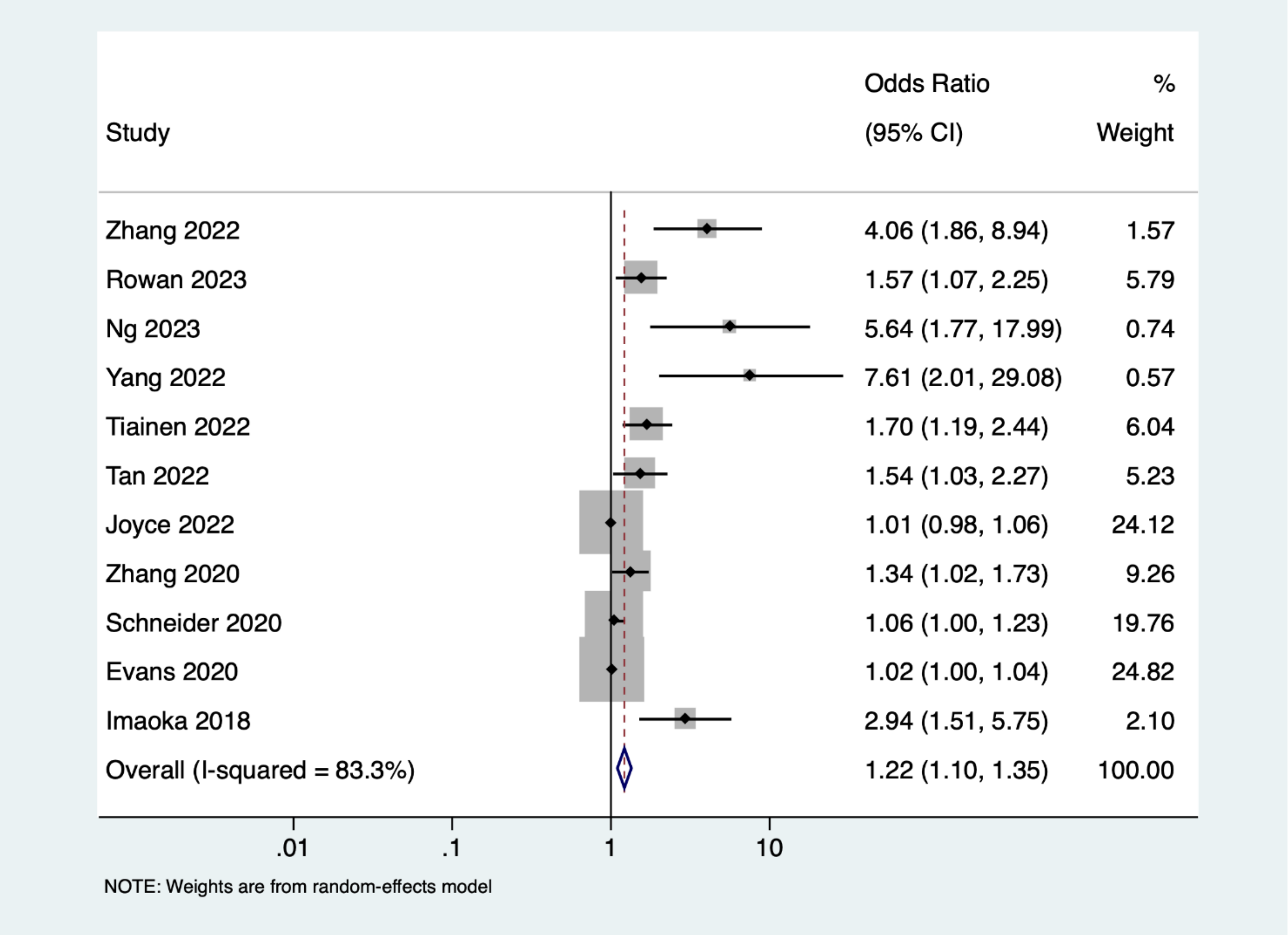

Supplementary file 6: Forest plot showing the association between frailty and poor functional outcome among stroke patients grouped by continent (adjusted)

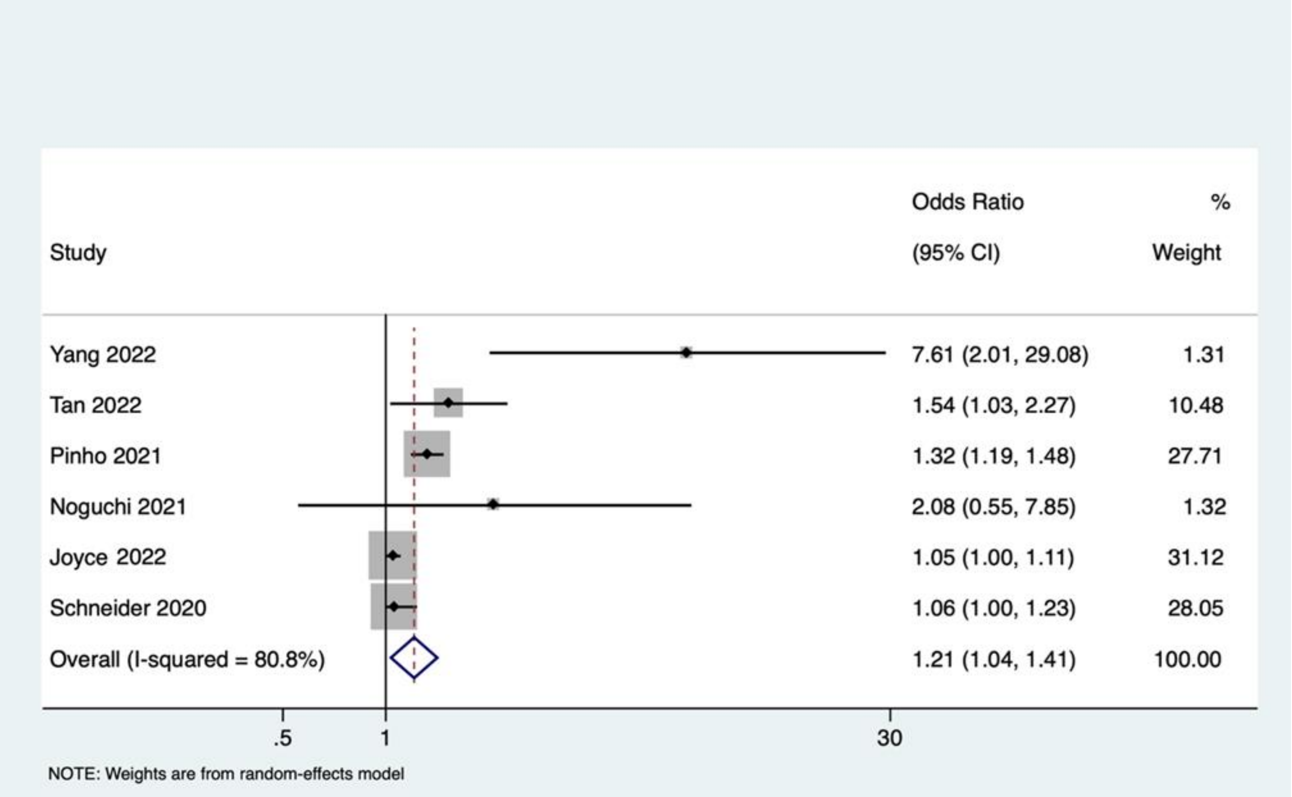

Supplementary file 7: Forest plot showing the association between frailty and mortality among stroke patients grouped by continent (adjusted)

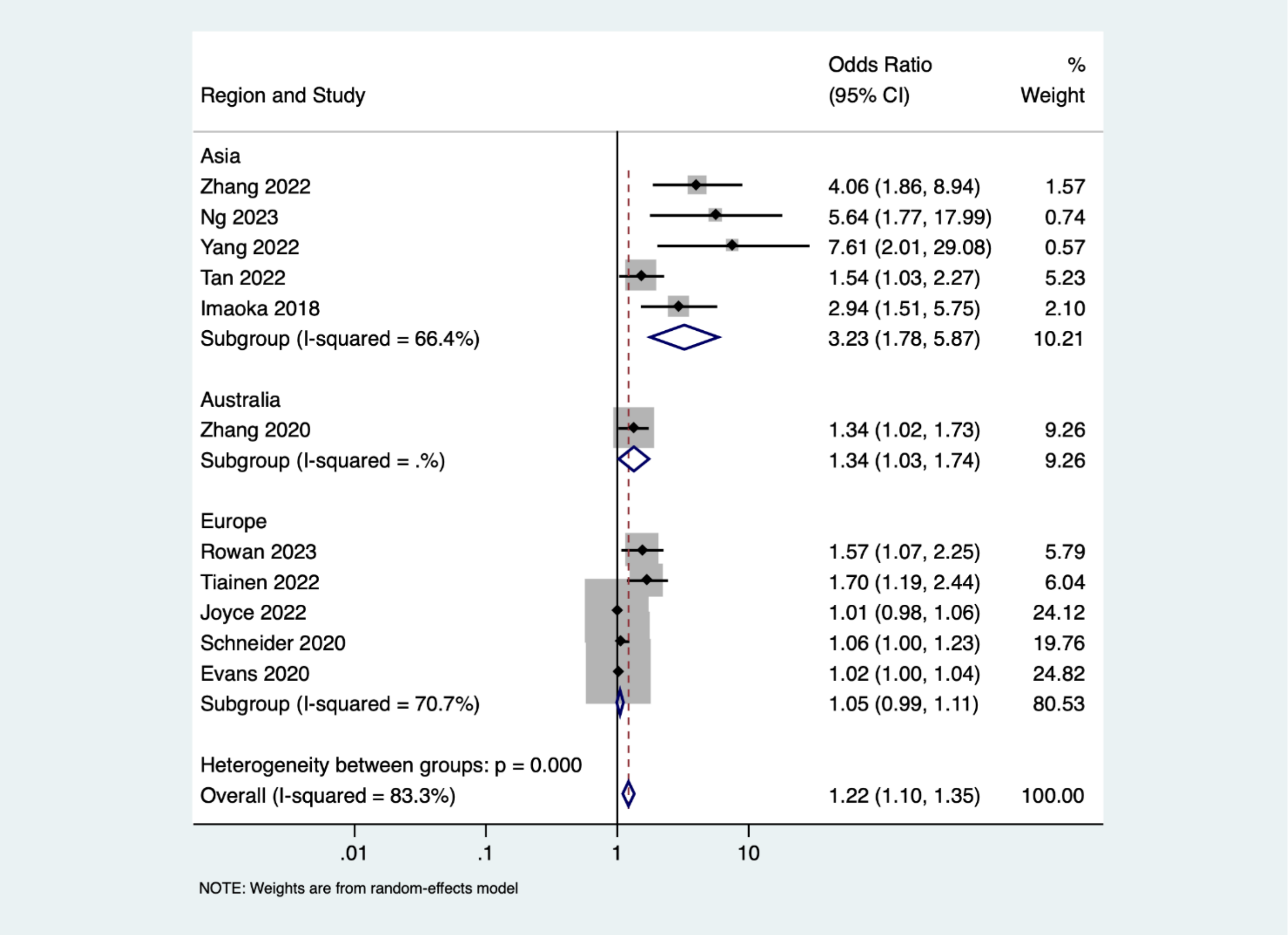

Supplementary file 8: Forest plot showing the association between frailty and poor functional outcome among stroke patients grouped by continent (adjusted)

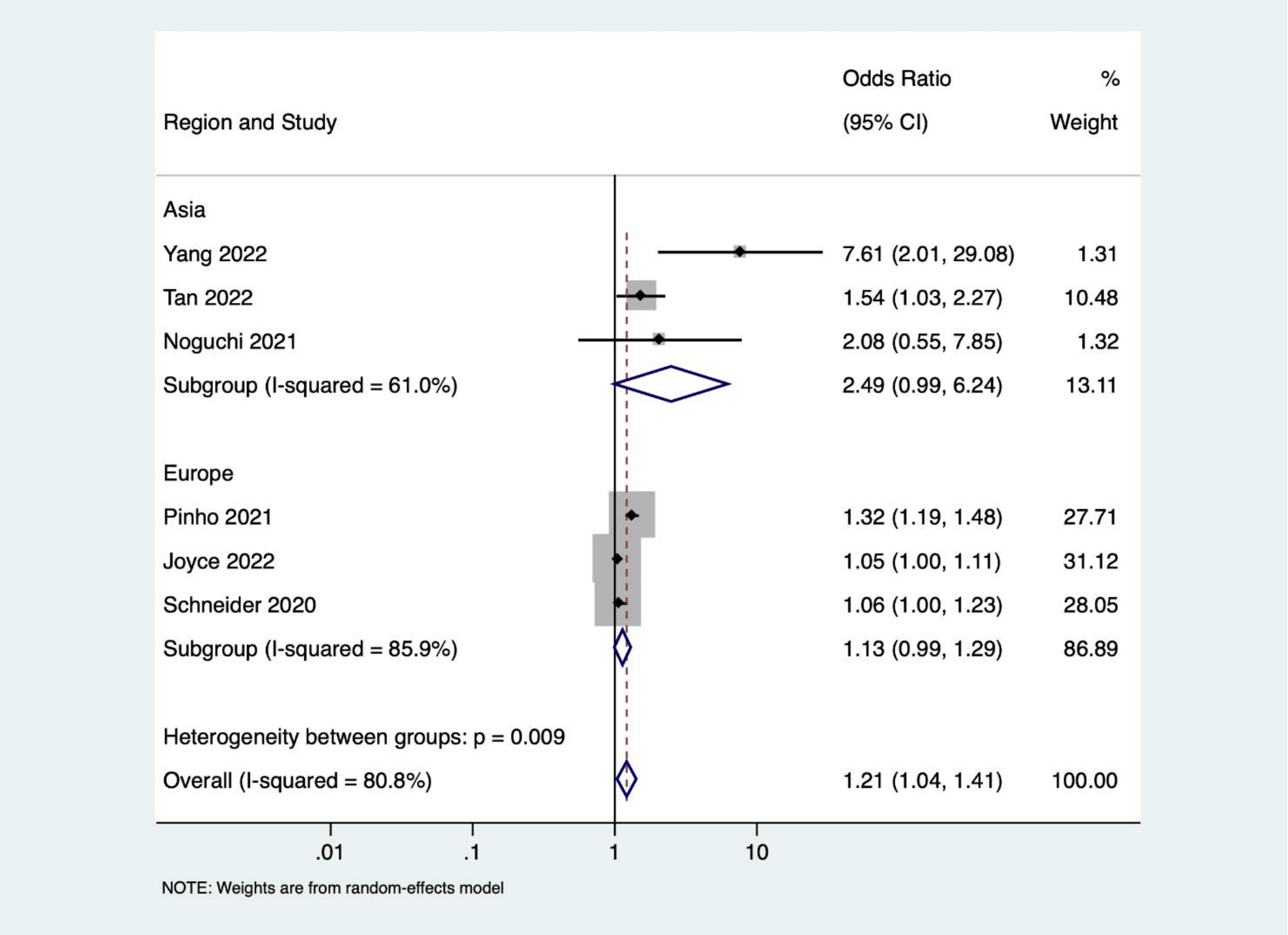

Supplementary file 9: Funnel plot showing the publication bias for studies reporting mortality (unadjusted)

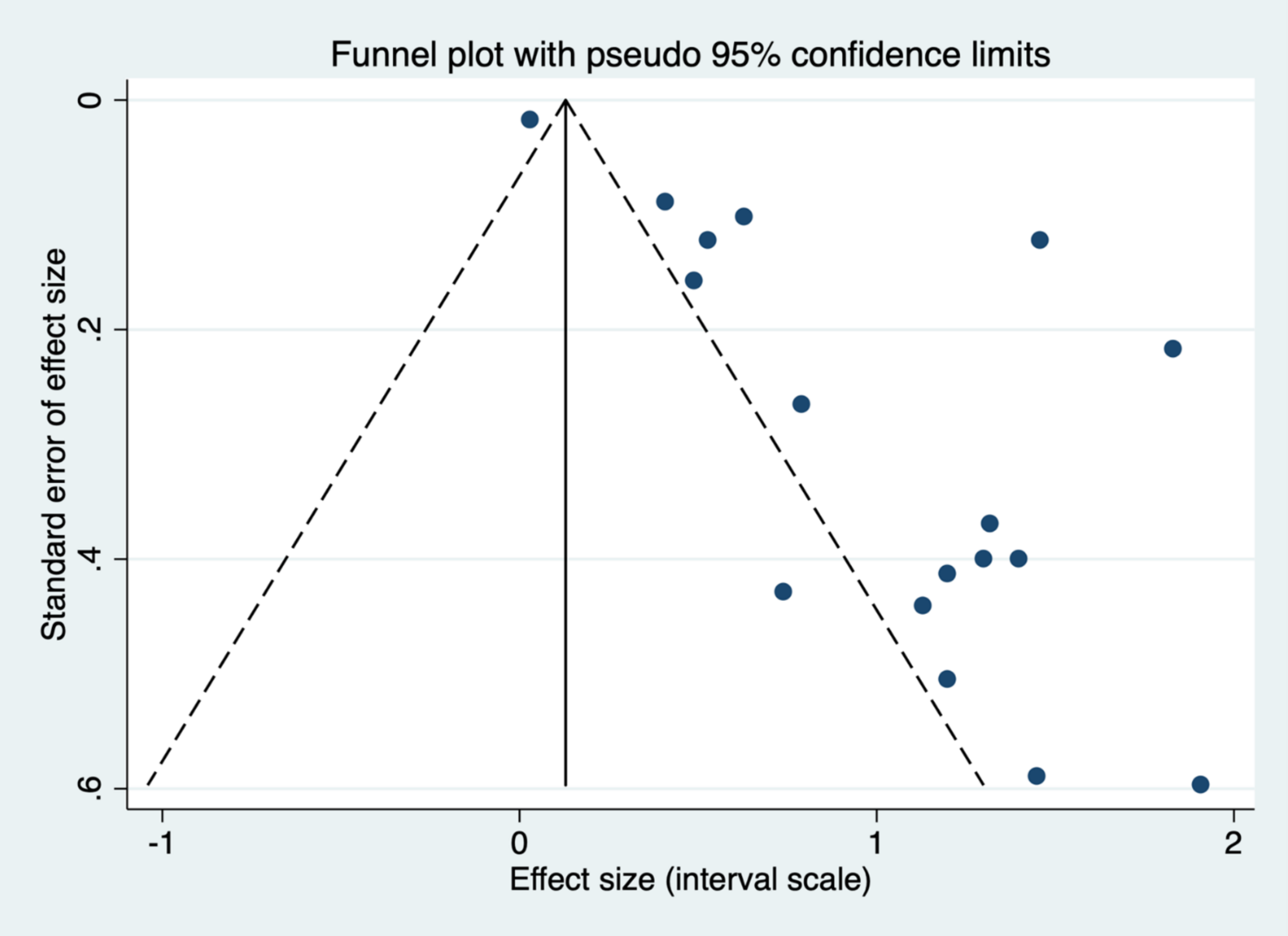

Supplementary file 10: Funnel plot showing the publication bias for studies reporting mortality (adjusted)

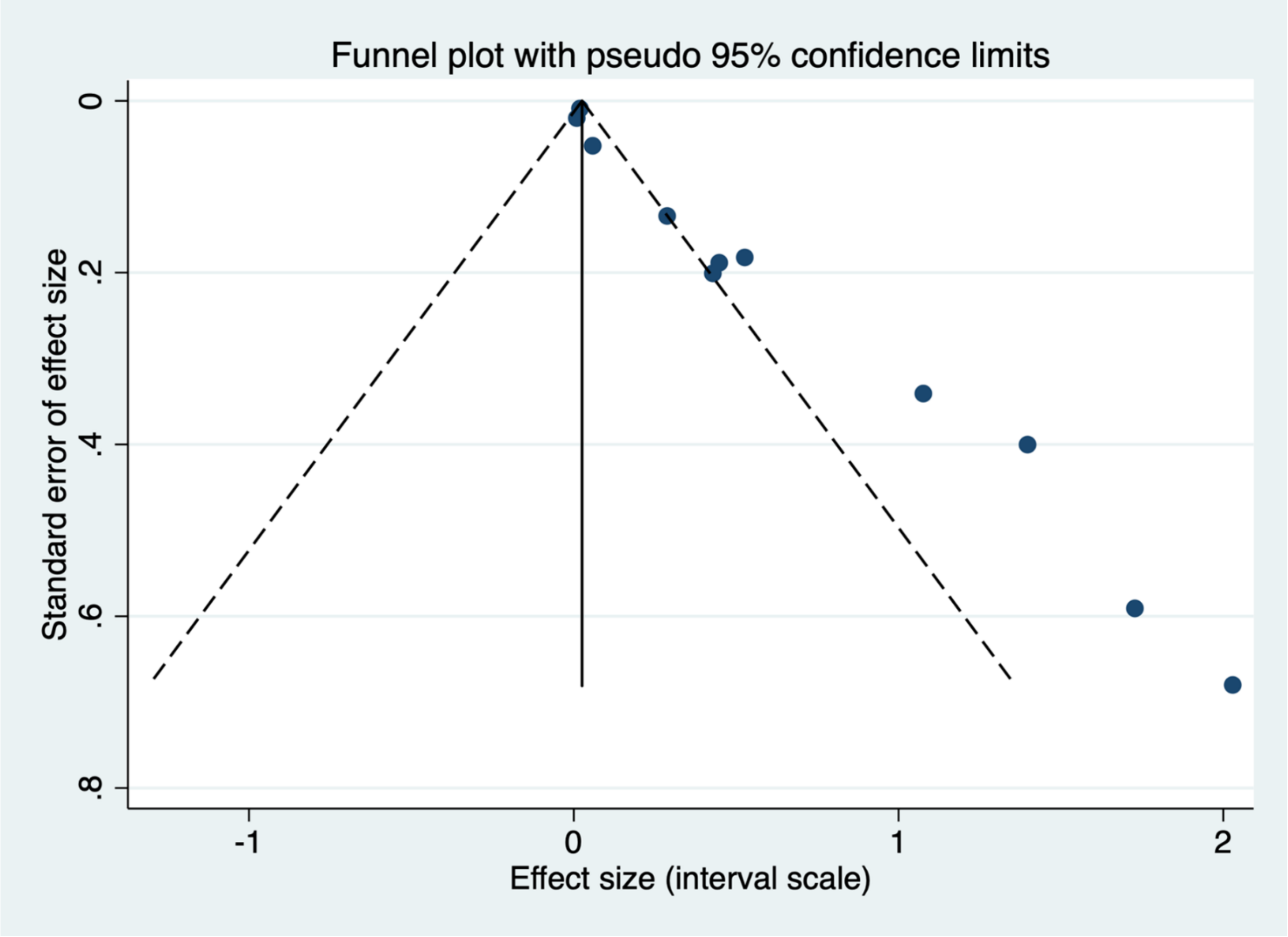

Supplementary file 11: Forest plot showing the prevalence of frailty among stroke patients sorted by quality of studies

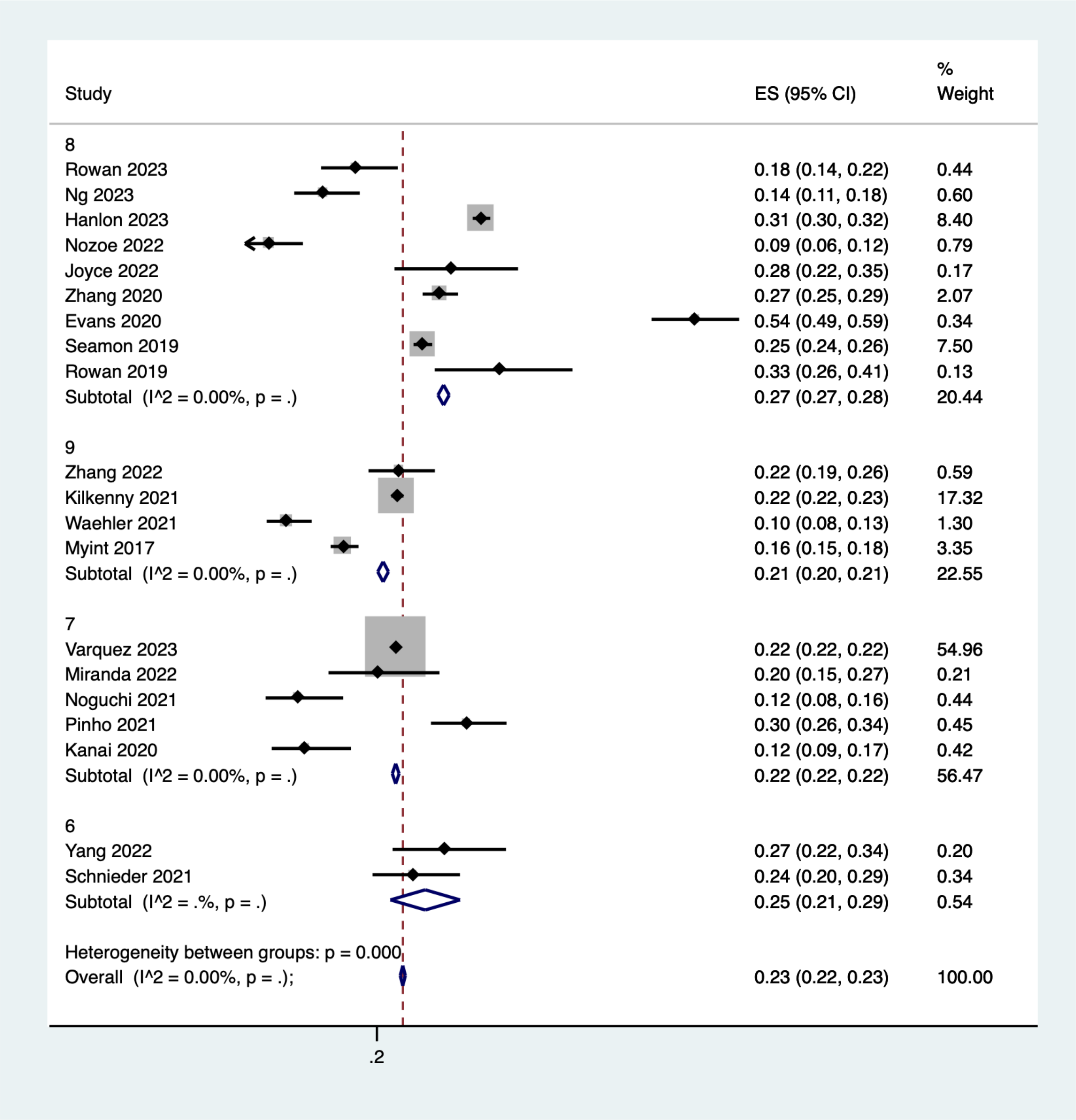

Supplementary file 12: Forest plot showing the association between frailty and mortality among stroke patients sorted by quality of studies [unadjusted]

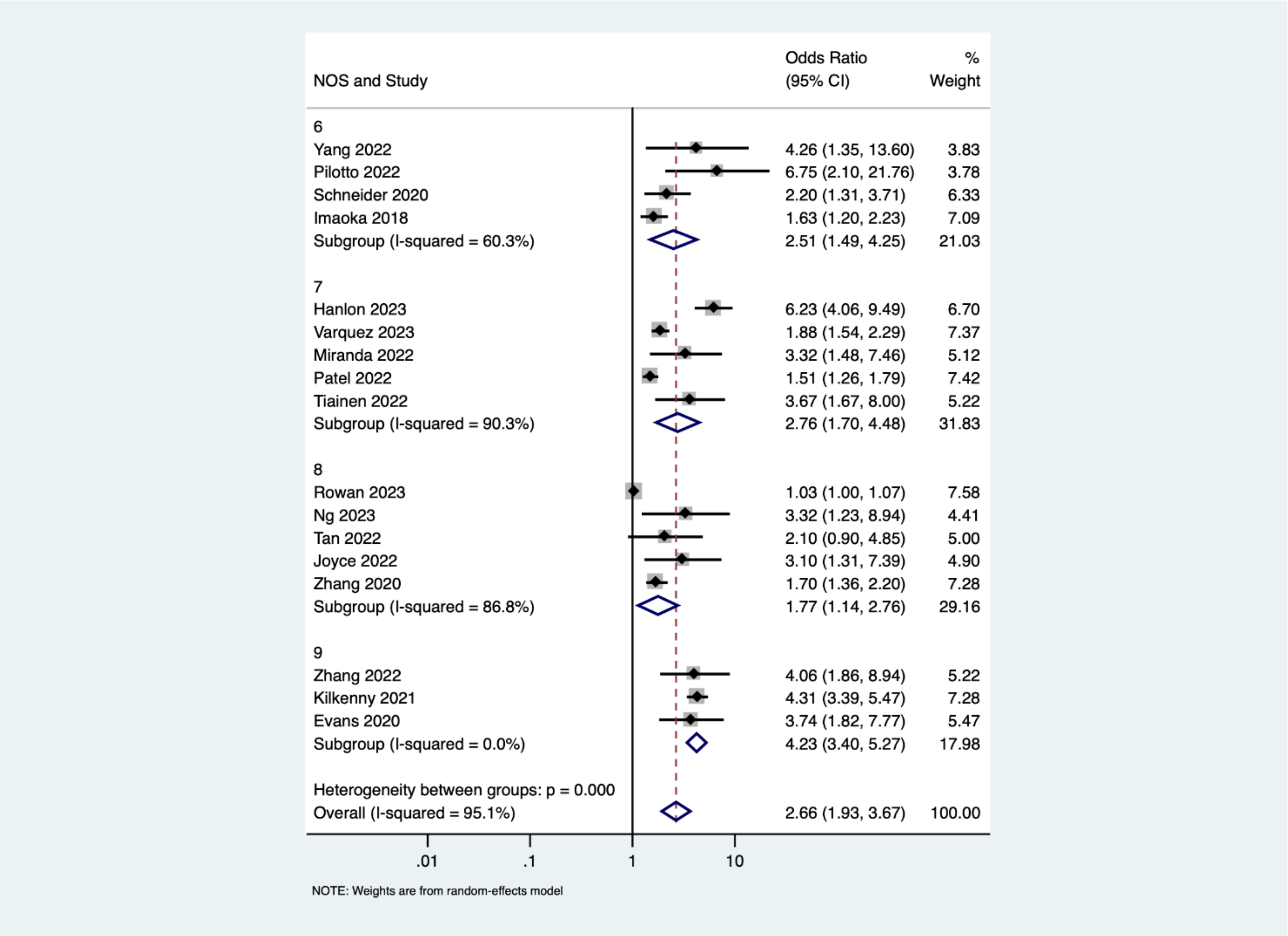

Supplementary file 13: Forest plot showing the association between frailty and poor functional outcome among stroke patients sorted by quality of studies [unadjusted]

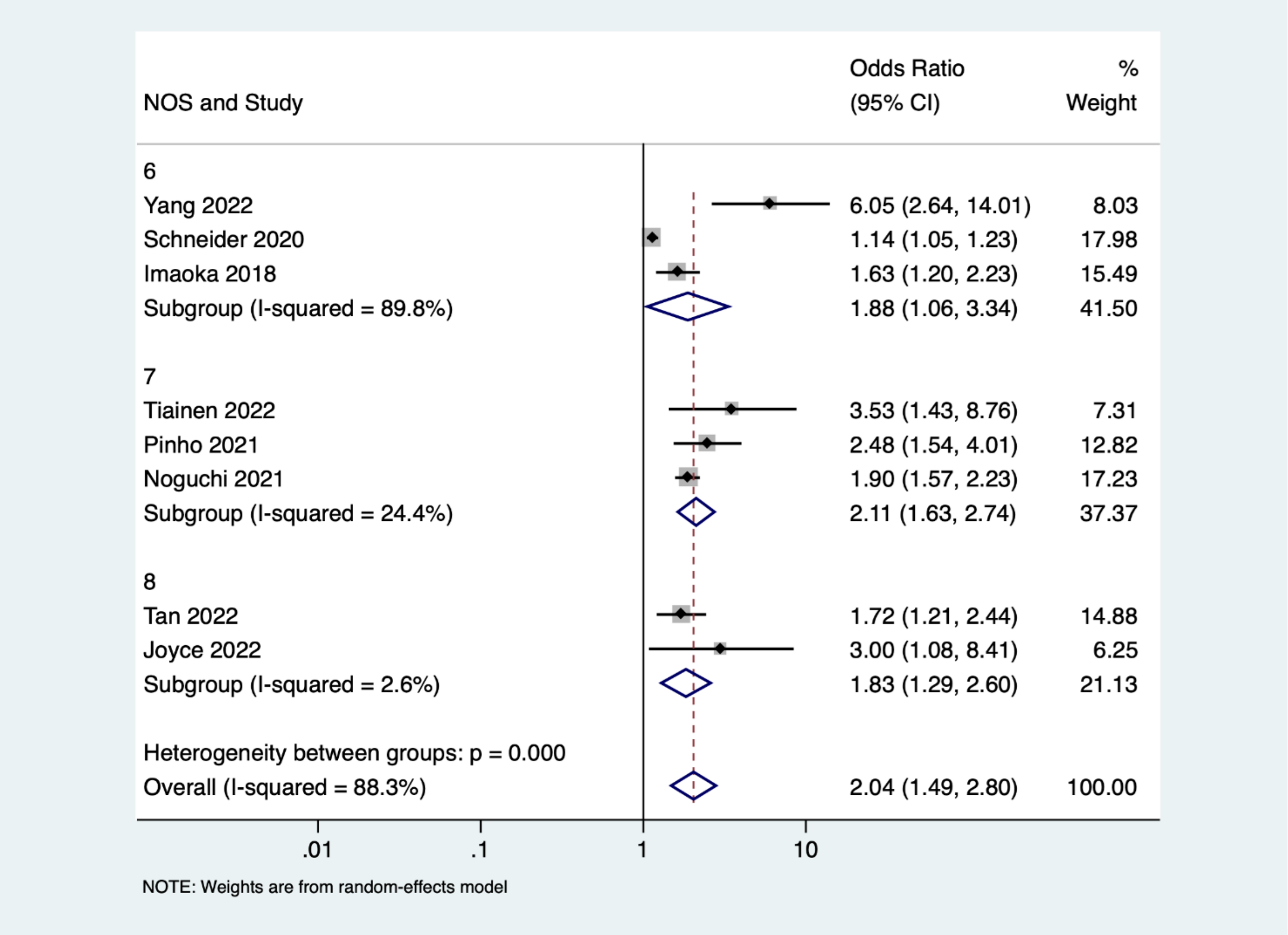

Supplementary file 14: Forest plot showing the association between frailty and mortality among stroke patients sorted by quality of studies [adjusted]

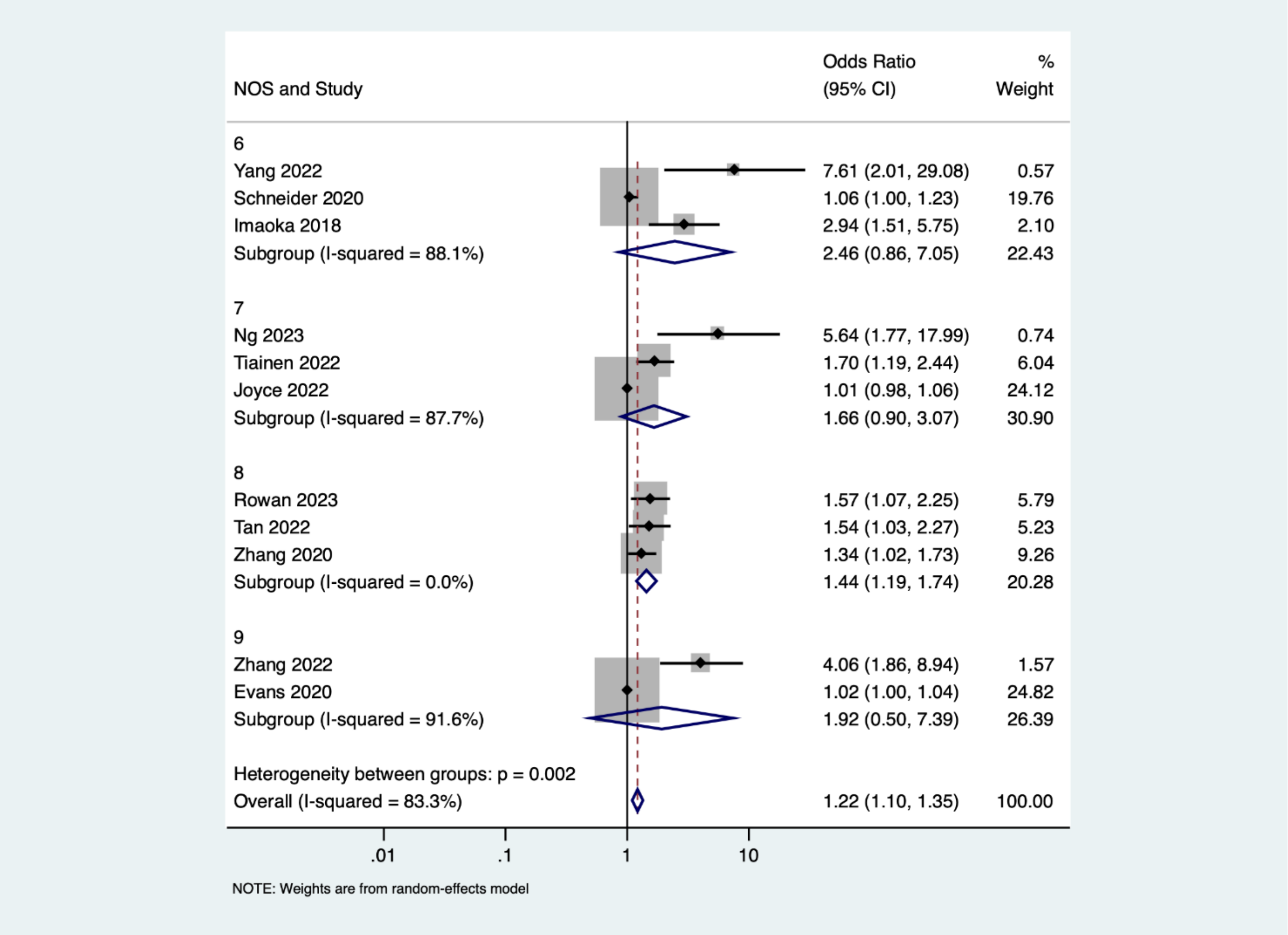

Supplementary file 15: Forest plot showing the association between frailty and poor functional outcome among stroke patients sorted by quality of studies [adjusted]

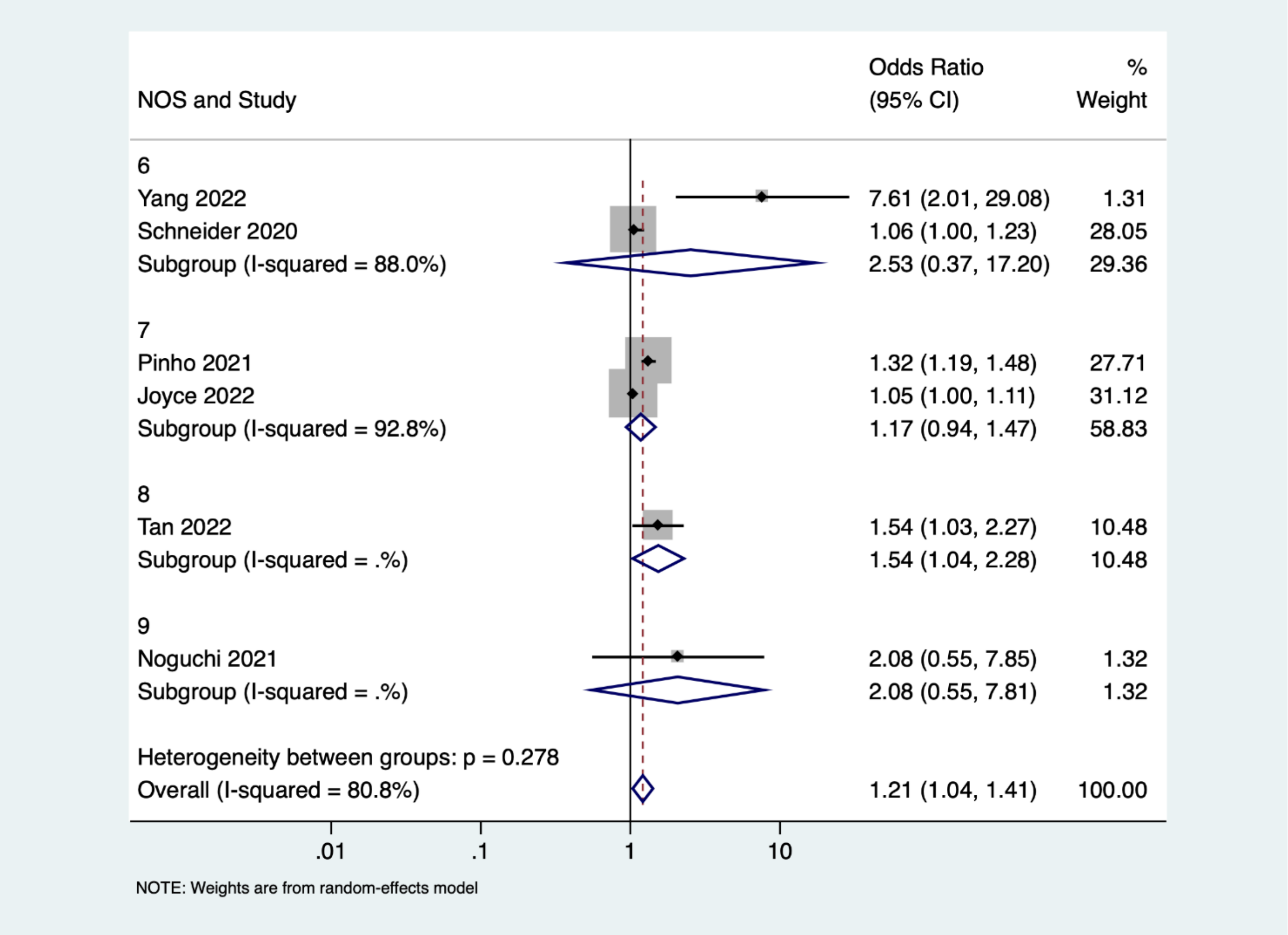

Supplementary file 16: Forest plot showing the prevalence of frailty among stroke patients grouped by tool used

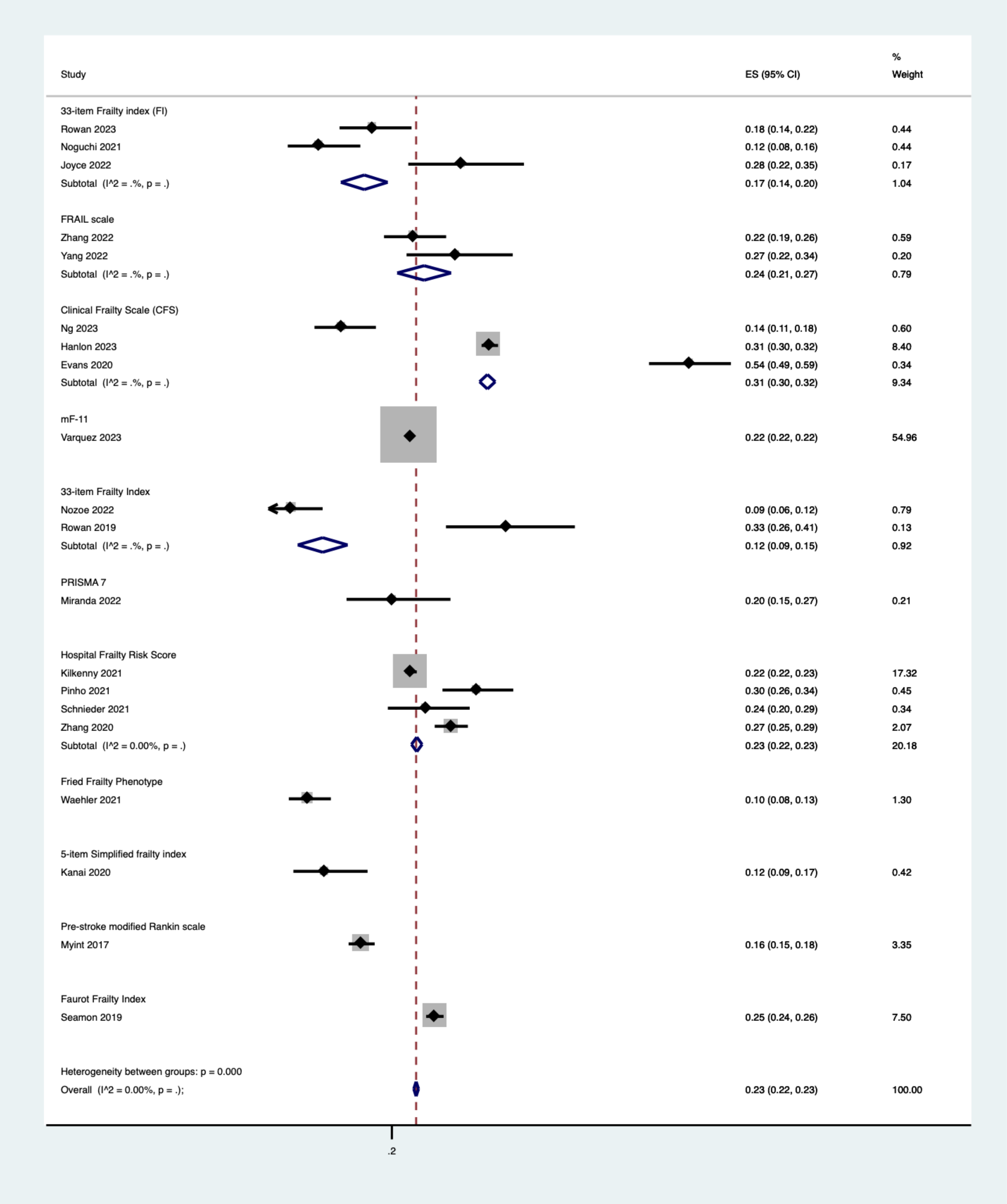

Supplementary file 17: Forest plot showing the comparison of hospital stay across frail and non-frail stroke cases

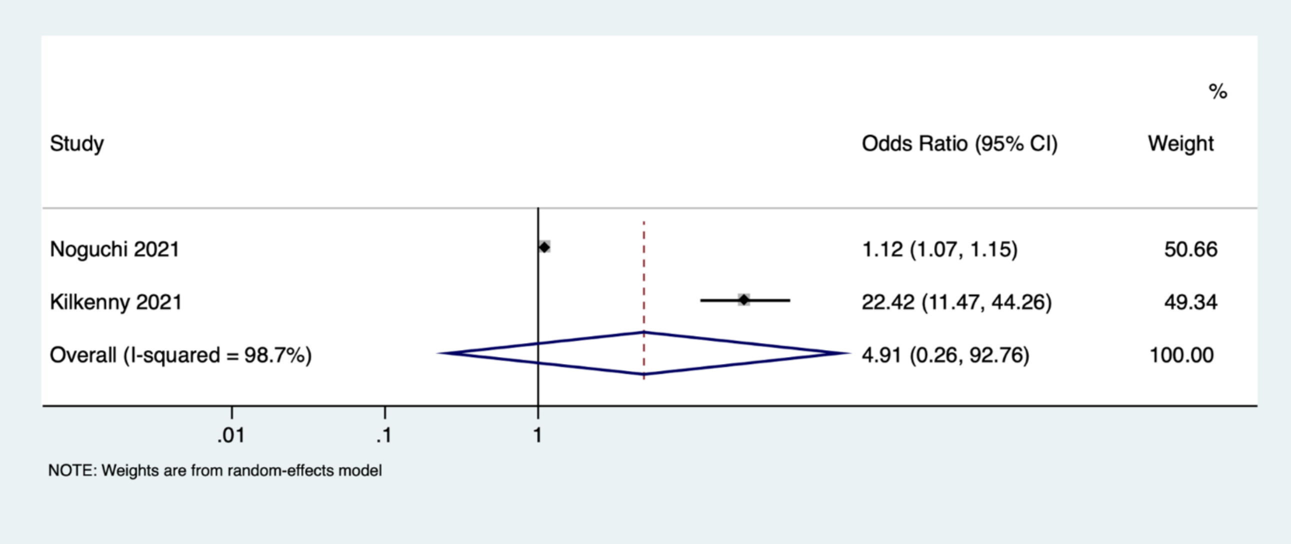

Supplementary file 18: Subgroup analysis based on the study design of the included studies for unadjusted mortality estimates

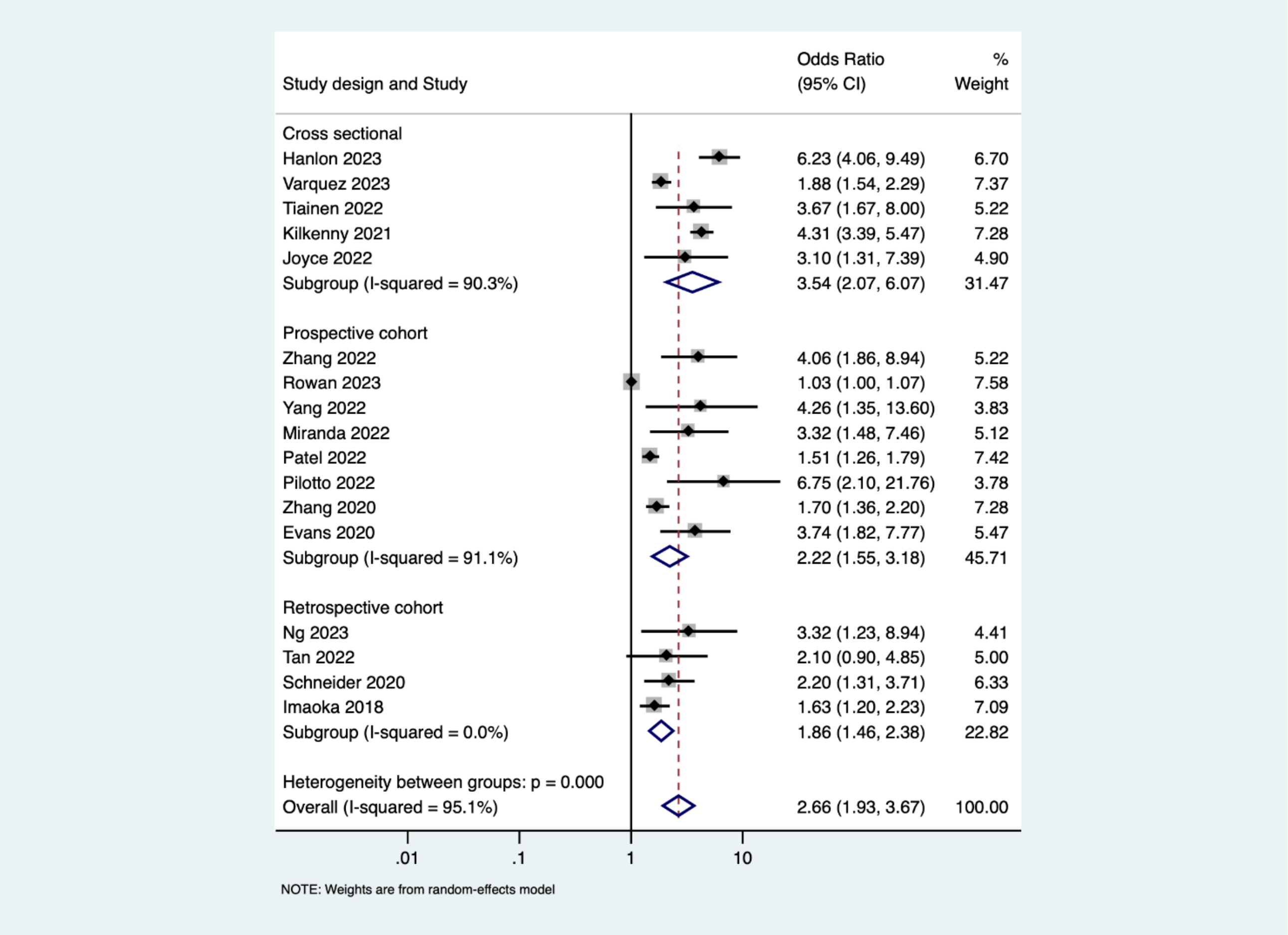

Supplement: Supplementary file 1 [file DataSheet_1.pdf]
